# Hybrid Nature-Inspired Optimization for the Cell Formation Problem with Machine Reliability and Alternative Routings

**DOI:** 10.3390/biomimetics11060387

**Published:** 2026-06-01

**Authors:** Paulo Figueroa-Torrez, Broderick Crawford, Orlando Durán, Martín Jurado-Camacho, Dayana Roxana Andrade Roque, Adrian Vargas-Gutierrez, Felipe Cisternas-Caneo

**Affiliations:** 1 Departamento de Ciencias Industriales, Medio Ambiente y Energía, Universidad Católica Boliviana “San Pablo”, Colón 734, Tarija, Bolivia; mjurado.c@ucb.edu.bo (M.J.-C.); dayana.andrade@ucb.edu.bo (D.R.A.R.); adrian.vargas.g@ucb.edu.bo (A.V.-G.); 2 Escuela de Ingeniería Informática, Pontificia Universidad Católica de Valparaíso, Avenida Brasil 2241, Valparaíso 2362807, Chile; felipe.cisternas.c@mail.pucv.cl; 3 Escuela de Ingeniería Mecánica, Pontificia Universidad Católica de Valparaíso, Valparaíso 2340025, Chile; orlando.duran@pucv.cl

**Keywords:** cell formation problem, machine reliability, alternative routing, black widow optimizer, golden eagle optimizer, metaheuristics hybridization

## Abstract

The Cell Formation Problem plays a fundamental role in cellular manufacturing due to its impact on efficiency, flexibility, and reliability. Its complexity increases under real-world conditions involving alternative process routes and machine reliability constraints, leading to the Generalized Cell Formation Problem with machine reliability. Researchers have classified the Cell Formation Problem as an NP-Hard problem. To address this computational complexity, this study presents a comparative and hybrid evaluation of the Black Widow Optimizer and the Golden Eagle Optimizer for the Generalized Cell Formation Problem with machine reliability, examining whether mechanisms derived from the Black Widow Optimizer can enhance the search behavior of the Golden Eagle Optimizer. The Black Widow Optimizer provides strong intensification through procreation, cannibalism, and mutation mechanisms, whereas the Golden Eagle Optimizer provides a balanced search process through its cruise and attack strategies. Experimental results show that the Black Widow Optimizer achieved better individual performance than the Golden Eagle Optimizer, with average RPD values of 0.855% and 1.068%, respectively. However, the hybrid strategy based on incorporating the mutation mechanism into the Golden Eagle Optimizer produced the best result, reaching an RPD of 0.592%. The study also employed the Wilcoxon–Mann–Whitney statistical test to validate the performance differences among algorithms, and the respective Big-O computational complexity was calculated. These findings highlight the potential of hybrid metaheuristics for designing robust and efficient manufacturing systems.

## 1. Introduction

The Cell Formation Problem (CFP) has become a central element in the design of cellular manufacturing systems [1] due to its direct impact on production efficiency, flexibility, and reliability. In real-world contexts, the complexity of the CFP increases considerably when alternative process routes for parts and constraints associated with machine reliability [2], such as Mean Time Between Failures (MTBF) [3], are incorporated, leading to the Generalized Cell Formation Problem with Machine Reliability (GCFP-MR). These factors bring the problem closer to industrial reality, where interruptions and the need for alternative routes are frequent, and their proper management is essential to maintain sustainable production performance.

In recent decades, research on the CFP has expanded from traditional approaches based on mathematical programming to the use of metaheuristic algorithms [4], which are recognized for their ability to generate high-quality solutions within reasonable computational times, especially for problems of an Non-deterministic Polynomial-time Hard (NP-Hard) nature [5]. For this purpose, two bio-inspired metaheuristics are considered: Black Widow Optimizer (*BWO*) [6] and Golden Eagle Optimizer (*GEO*) [7]. Both algorithms stand out for their ability to address complex optimization problems.

The *BWO* is inspired by the behavior of the black widow spider, using elimination and selective reproduction mechanisms that enhance diversity and exhibit fast convergence. The *GEO*, on the other hand, replicates the hunting strategy of golden eagles, balancing exploration and intensification through guided and adaptive movement in the search space [8]. Taken together, these two behaviors show a natural complementarity for the GCFP-MR: the *GEO* promotes broad exploration by mirroring high-altitude flight and patrolling over large regions of the search space, whereas the *BWO* strengthens diversification and helps avoid premature convergence through mutation and procreation operators that aggressively perturb parts of a solution, while selection and cannibalism mechanisms retain the most promising configurations.

The growing interest in these algorithms reflects a paradigm shift: there has been a transition from simplified formulations, mainly oriented toward theoretical analysis, to more realistic, hybrid, and problem-oriented approaches [9]. Several studies have shown that hybrid metaheuristic approaches can effectively solve the classical CFP, particularly through combinations of genetic algorithms and local search strategies aimed at improving grouping efficacy [10]. However, these contributions are largely restricted to deterministic environments with fixed process routes and do not account for machine reliability. Beyond reliability-oriented formulations, other extensions of the CFP have focused on incorporating additional dimensions, such as the simultaneous assignment of parts, machines, and workers in the Cubic Cell Formation Problem (CCFP), where hybrid Genetic Algorithm (GA) and Simulated Annealing (SA) approaches have been used to address workforce skills and quality-related objectives. Nevertheless, these studies prioritize human and quality aspects rather than machine reliability and alternative routings [11].

Addressing the GCFP-MR is relevant because cell configurations that ignore route flexibility and machine reliability may lead to solutions that are optimal only under idealized conditions but less effective in real production environments. By jointly considering alternative routings and reliability-related constraints, the problem supports the design of manufacturing systems that are more robust to machine failures, production interruptions, and operational variability. In this context, metaheuristic approaches are particularly suitable because they can explore large combinatorial search spaces and generate high-quality solutions within reasonable computational times [12].

In this context, the present article aims to evaluate the performance of the *GEO*, the *BWO*, and their hybridization in solving the GCFP-MR. The novelty of this study lies in the comparative and hybrid evaluation of these two bio-inspired metaheuristics for a reliability-oriented cell formation problem with alternative routings. Unlike previous studies that focused on applying a single metaheuristic to this problem, this work examines whether the exploration mechanism of the *GEO* can be strengthened by incorporating *BWO*-based operators, particularly mutation, procreation, and cannibalism mechanisms.

**Figure 1:**
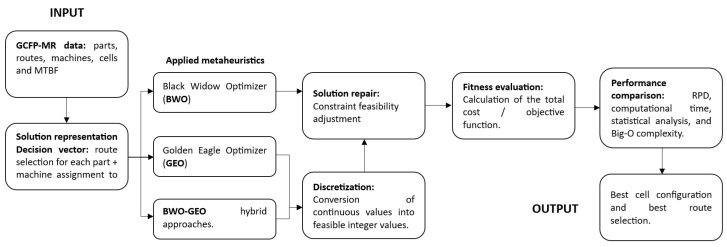
Conceptual framework of the proposed metaheuristic approach for solving the GCFP-MR.

The fundamental premise is that the global exploration capability of the *GEO*, when combined with the mutation-driven ability of the *BWO* to move away from local optima, can produce superior solution quality compared to the individual algorithms. To achieve this objective, the article is structured as follows: Section 2 reviews the relevant literature on metaheuristics in the GCFP-MR; Section 3 describes the methodology and experimental configuration; Section 4 presents the results and discussion; Section 5 provides the statistical analysis; and Section 6 concludes with final observations and guidelines for future research. In addition, Figure 1 provides a conceptual overview of the proposed approach, summarizing its main stages.

## 2. Theoretical Background

This section reviews the evolution of the CFP, beginning with its origins in Group Technology (GT) [13] and progressing toward formulations that incorporate alternative routing [14] and machine reliability [15]. The discussion analyzes the development of conceptual, mathematical, and algorithmic approaches that have shaped the field, leading to the current state of the art. Additionally, this section examines the metaheuristic strategies that have been proposed to address the CFP and its generalized variants, highlighting the main contributions, methodological advances, and hybrid frameworks employed to address the complexity of these manufacturing system design problems.

### 2.1. The Origin of the Cell Formation Problem

The theoretical background of the CFP originates from the concept of GT, which establishes that parts that share similar design characteristics and processing requirements should be grouped into families. Such grouping enables the reuse of technological information and reduces unnecessary variation in production processes [16].

Burbidge [13] expanded the initial ideas of GT and developed the Production Flow Analysis (PFA) method, which facilitates the analysis of production flow and the reorganization of machines based on similarities in the operations required by the parts. Through this method, the concept of machine families was formally introduced, referring to the grouping of machines that perform similar or complementary operations on specific sets of parts. This approach supports the formation of more coherent and efficient manufacturing cells aligned with the technological routes of the parts [17]. The CFP was subsequently formalized as a structured grouping problem when explicit methods were proposed to classify machines according to the parts they process, establishing the first conceptual models for grouping machine components [13].

### 2.2. Incorporation of Alternative Routes into the Cell Formation Problem

The incorporation of alternative routes into the CFP represented a significant advancement in the literature, as it acknowledges that a single part can be manufactured through multiple machine sequences [18]. This flexibility enhances the adaptability of the production system; however, it also increases the combinatorial complexity of the problem due to the larger number of feasible machine–part assignments. Such combinatorial growth leads the CFP, particularly when alternative routes are included, to be classified as an NP-hard problem [19,20], since the number of possible configurations grows exponentially with the number of parts, machines, and available routes [21].

In manufacturing environments, a part may be processed through more than one feasible process plan, meaning that multiple operation sequences and processing routes can lead to the same finished product. These alternative production possibilities are commonly referred to as alternative routes [22]. These studies consolidate the transition from the classical CFP to the Generalized Cell Formation Problem (GCFP), demonstrating that alternative routes not only enhance manufacturing flexibility but also align the model more closely with the operational characteristics of modern industrial environments.

### 2.3. Cell Formation Problem with Machine Reliability

In the CFP, integrating machine reliability is essential for designing cells that are able to operate under real conditions. One of the first works to address this approach was presented by Sofianopoulou [15], who proposed a model combining alternative routes and reliability, demonstrating that machine failures significantly modify the optimal structure of cells.

In line with these advances, Karoum and Elbenani [23,24] developed a model that integrates failure rates and repair times into machine–part allocation, allowing for the formation of cells that are more robust in the face of unexpected events. Their approach demonstrates that a CFP with these considerations is no longer a deterministic problem and can be addressed under operational uncertainty.

More recently, Figueroa-Torrez et al. [25] used a model that simultaneously integrates alternative routes and machine reliability associated with MTBF, demonstrating that reliability not only increases the quality of cellular design but also its viability in real industrial environments. These contributions show that machine reliability transforms the CFP into a more comprehensive and realistic problem, where the objective is not only to efficiently group machines and parts, but also to ensure that cells maintain operational continuity considering machine failures. This approach has demonstrated that including reliability not only improves the accuracy of solutions, but also makes them more viable in real manufacturing environments.

### 2.4. Metaheuristics Applied to the Generalized Cell Formation Problem with Machine Reliability Considerations

Considering that the GCFP-MR has an NP-Hard nature, as mentioned by Shirzadi et al. [19], it is not recommended to use exact methods, as they become impractical for more realistic instances. A metaheuristic generally refers to approximate optimization algorithms that are not designed for a specific problem but provide a flexible approach applicable to a wide range of domains [26]. These are non-exact methods that integrate mechanisms for exploring and exploiting the search space to obtain high-quality solutions within acceptable computational times [27].

Although many metaheuristics were originally proposed for continuous search spaces, their application is not necessarily restricted to continuous optimization problems. In combinatorial problems such as the GCFP-MR, where several decisions can be represented using discrete variables, continuous metaheuristics may also be useful when appropriate transformation mechanisms are incorporated. In this regard, Crawford et al. [28] emphasize that adapting continuous metaheuristics to binary search spaces allows researchers to take advantage of their search capabilities in problems whose feasible solutions are encoded through zero-one variables.

Among the first applications, GA [29], SA [30] and Tabu Search (TS) [31] stand out. Over time, more sophisticated methods emerged, such as *BWO*, introduced by Hayyolalam and Kazem [6]. This algorithm, based on the reproductive behavior of black widows, balances exploration and exploitation, avoids falling into local optima, and achieves fast convergence toward optimal solutions.

In the specific context of the GCFP-MR, Jabalameli et al. [32] addressed the problem considerations by applying SA, GA, and a Memetic Algorithm (MA). Later, Jouzdani et al. [33] proposed a generalized model that incorporated machine reliability, material handling costs, setup costs, and part demands, solving it using a modified SA. Subsequently, Karoum and Elbenani [34] introduced a modified Clonal Selection Algorithm (CSA) with a local search mechanism to improve solution quality and computational efficiency. These studies represent early efforts to apply metaheuristic methods to more realistic CFP involving reliability-related decisions.

Figueroa-Torrez et al. [25] adapted the *BWO* to the binary domain, naming it Binary Black Widow Optimizer (B-BWO), to solve the GCFP-MR, demonstrating its effectiveness compared to other metaheuristics. However, the authors caution that binary encoding may not fully reflect the discrete nature of the CFP. In addition, the computational cost is higher, as a larger solution vector leads to an expanded combinatorial space.

Recent studies have also reinforced the applicability of continuous metaheuristics to combinatorial problems. For instance, Crawford et al. [35] proposed a discrete version of the Pufferfish Optimization Algorithm (POA), showing that continuous search mechanisms can be adapted to discrete problems through the combination of transfer functions and repair rules. Based on this, recent research explores new discrete strategies, such as the *GEO*, introduced by Mohammadi-Balani et al. [7], which is inspired by the hunting behavior of golden eagles. This algorithm combines phases of global exploration (search) and local exploitation (attack), adapting the speed and direction of flight to improve convergence.

#### Application with Hybrid Metaheuristics

Metaheuristics have developed significantly in recent decades, especially when researchers recognized the performance limitations of algorithms that rely on a single strategy to address complex combinatorial problems. As noted by Blum et al. [26], early developments in metaheuristics progressed with limited interaction with traditional operations research, which resulted in an underutilization of well-established optimization principles. When purely heuristic strategies began to reach their performance limits, the integration of complementary algorithmic mechanisms emerged as a natural and effective research direction.

Hybrid metaheuristics seek to combine the strengths of different optimization strategies, typically balancing exploitation and exploration to improve solution quality and robustness. Evolutionary algorithms play a central role in this context due to their flexible structure, which allows the inclusion of specialized operators aimed at reinforcing either exploitation or exploration. According to Máximo and Nascimento [36], hybrid schemes that incorporate evolutionary perturbation mechanisms within local search have achieved competitive performance, demonstrating the advantage of exploiting complementary behaviors within a unified framework.

Additionally, recent reviews such as Velasco et al. [37] highlight that many of the most influential metaheuristics introduced over the last two decades integrate hybrid principles, including adaptive parameter control, specialized evolutionary operators, and mechanisms to prevent stagnation in local optima. These findings show that hybridization is not only a recurring trend but also a promising direction for developing more effective optimization tools.

Considering the complexity of the GCFP-MR, characterized by alternative process routes, machine reliability, and highly interdependent combinatorial constraints, the application of hybrid metaheuristics becomes particularly relevant. These approaches provide a structured mechanism to balance exploration and exploitation across different regions of the solution space, enhancing robustness when dealing with uncertainty and variability in manufacturing environments. As shown by Golmohammadi et al. [38], hybrid methodologies that integrate complementary algorithmic components are particularly effective for problems related to alternative routing and machine reliability, reinforcing their ability to address the structural challenges present in the GCFP-MR.

In a related cell formation context, Golmohammadi et al. [39] proposed a hybrid metaheuristic algorithm based on GA, Keshtel Algorithm (KA), and Red Deer Algorithm (RDA) for the GCFP. In addition, Karoum and Elbenani [24] proposed a hybrid Cuckoo Search (CS) approach for the GCFP-MR. However, in this case, the hybridization does not combine two different metaheuristics; rather, it integrates a local search mechanism into the CS algorithm to improve exploitation and solution quality.

To complement the previous discussion and provide a clearer view of the current state of research, Table 1 summarizes selected studies related to GCFP-MR and metaheuristic solution methods. The table also distinguishes whether each contribution employs a standalone metaheuristic, a partial hybridization based on local search or algorithmic enhancement, or a hybrid strategy that combines complementary metaheuristic mechanisms.

As shown in Table 1, previous studies have incorporated relevant aspects such as alternative process routes, machine reliability, layout decisions, and different metaheuristic approaches. However, most contributions rely on standalone metaheuristics or partial hybridizations based on local search mechanisms. In contrast, the present study evaluates the individual performance of *BWO* and *GEO* and proposes hybrid variants that integrate mechanisms derived from *BWO* into *GEO*. This provides a more direct assessment of hybridization between complementary metaheuristic components for solving the GCFP-MR.

## 3. Methodology

### 3.1. Mathematical Model

The GCFP aims to be more realistic by incorporating key factors into cell formation. One of these is the presence of processes that need machines in different cells, creating intercellular movement [40], which involves a cost associated with moving between cells. Another relevant factor is the possibility of machine failures, which interrupt the processes in the affected routes [22]. To estimate the impact of these failures, a cost is associated with each failure that occurs. Both elements are considered in the model by Saeed Jabal Ameli and Arkat [41], which determines intercellular costs according to the quantity and value of the parts that undergo such movements, and the breakdown cost based on the cost associated with the number of failures of each machine.

### 3.2. Model Assumptions

The GCFP works with the following premises:

- The total number of cells is known.
- The lower and upper limits of machines per cell are known.
- Each part has at least one process route, but only a single route can be selected.
- Each route has different operations, performed by machines, with ordered sequences.
  The sequence helps to determine when a part passes from one cell to another.
- Each type of part has its processing time on each machine.
- Multiple identical machines are not considered.
- The production demand of each part is known and deterministic.
- MTBF is used for the machine reliability calculation.

### 3.3. Classic Mathematical Model

#### 3.3.1. Parameters

The parameters considered are:

*m*: Quantity of machines;
*n*: Quantity of parts;
*c*: Quantity of cells;
*P_i_*: Production volume for part *i*;
*q_i_*: Quantity of routes per part *i*;
*L_l_*: Lower limit of the quantity of machines in cell *l*;
*U_l_*: Upper limit of the quantity of machines in cell *l*;
*K_ij_*: Quantity of machines in route *j* of part *i*;
  $u_{ij}^{1}$, $u_{ij}^{2}$,…, $u_{ij}^{k_{ij}}$: Machine indices of route *j* of part *i*;
*T_ik_*: Duration required for processing a part *i* on machine *k*;
*B_k_*: Breakdown cost associated with machine *k*;
*IMC_ij_*: Intercellular movement cost for part *i* in route *j*;
*MTBF_k_*: Mean time between failure of machine *k*.

#### 3.3.2. Decision Variables of the Model

The decision variables are:

vmkt=1, if furnace m produces product k in period t 0, otherwise 

Zij=1, if route j of part i is selected 0, otherwise Ykl=1, if machine k is located in cell l 0, otherwise Xijklsl=1, if route j of part i is selected, machine k is located in cell l and machine s is not located in cell l 0, otherwise

#### 3.3.3. Objective Function

The mathematical model used by Saeed Jabal Ameli and Arkat [41] is presented below:

(1) $$\min\;TC=\sum_{i=1}^{n}\sum_{j=1}^{q_{i}}\sum_{k=1}^{K_{ij}-1}\sum_{l=1}^{c}\;IMC_{ij}\;P_{i}\;X_{ij\left(u_{ij}^{k_{ij}}\right)l\left(u_{ij}^{k_{ij}+1}\right)l}+\sum_{i=1}^{n}\sum_{j=1}^{q_{i}}\sum_{k=1}^{K_{ij}}\;Z_{ij}\;\frac{P_{i}\;T_{i(u_{ij}^{k_{ij}})}\;B_{\left(u_{ij}^{k_{ij}}\right)}}{MTBF_{\left(u_{ij}^{k_{ij}}\right)}}$$

Subject to:

(2) $$\sum_{j=1}^{q_{i}}Z_{ij}\;=\;1\;\;\forall i=1,2,\ldots ,n$$

(3) $$\sum_{l=1}^{c}Y_{kl}\;=\;1\;\;\forall k=1,2,\ldots ,m$$

(4) $$\sum_{k=1}^{m}Y_{kl}\;\leq \;U_{l}\;\;\forall l=1,2,\ldots ,c$$

(5) $$\sum_{k=1}^{m}Y_{kl}\;\geq \;L_{l}\;\;\forall l=1,2,\ldots ,c$$

(6) $$\begin{array}{cc} X_{ijklsl}\;\leq \;Z_{ij} & \;\forall i=1,2,\ldots ,n;\;\forall j=1,2,\ldots ,q_{i};\;\\ & \;\;\forall k,s=1,2,\ldots ,m;\;\forall l=1,2,\ldots ,c \end{array}$$

(7) $$\begin{array}{cc} & X_{ijklsl}\;\leq \;Y_{kl}\;\;\;\forall i=1,2,\ldots ,n;\;\forall j=1,2,\ldots ,q_{i};\;\\ & \;\;\;\;\;\;\;\;\forall k,s=1,2,\ldots ,m;\;\forall l=1,2,\ldots ,c \end{array}$$

(8) $$\begin{array}{cc} & X_{ijklsl}\;\leq \;\left(1-Y_{sl}\right)\;\;\forall i=1,2,\ldots ,n;\;\forall j=1,2,\ldots ,q_{i};\;\\ & \;\;\;\;\;\;\;\;\;\;\;\forall k,s=1,2,\ldots ,m;\;\forall l=1,2,\ldots ,c \end{array}$$

(9) $$\begin{array}{cc} Z_{ij}+Y_{kl}+\left(1-Y_{sl}\right)-X_{ijklsl}\leq 2 & \forall i=1,2,\ldots ,n;\;\forall j=1,2,\ldots ,q_{i};\;\\ & \;\;\;\forall k,s=1,2,\ldots ,m;\;\forall l=1,2,\ldots ,c \end{array}$$

(10) $$Z_{ij},Y_{kl},X_{ijklsl}\in \left\{0,1\right\}$$

The objective function (Equation (1)) consists of two terms. The first term calculates the total cost of intercellular movements, while the second term determines the total machine breakdown cost.

Equation (2) defines that each part can only select a single route. Equation (3) establishes that each machine must be assigned exclusively to one cell. Equations (4) and (5) set the maximum and minimum number of machines allowed per cell. Equation (6) determines that intercellular movement is activated exclusively by the route selected for a given part, ensuring that movements are not arbitrarily generated. Similarly, Equation (7) states that an intercellular movement can only occur if both the origin machine and its corresponding cell assignment have already been defined, preventing infeasible or undefined transfers. Furthermore, Equation (8) guarantees that the destination machine of an intercellular movement is assigned to a different cell from the source machine, thereby enforcing the intercellular nature of the movement. Finally, Equation (9) ensures that any intercellular movement originates from a machine located in a selected cell and is directed to another machine assigned to a different cell, preserving logical consistency between routing and cell formation decisions. According to Equation (10), all decision variables are binary.

### 3.4. Black Widow Optimizer

#### 3.4.1. Inspiration: Black Widows in Mating Season

During the mating season of the black widow spider, the female builds her web and marks specific areas to attract a male. Once a male enters the web, his presence repels other males from approaching. A distinctive aspect of this species’ reproductive behavior is that females may consume males during or after mating. When the young are born, cannibalistic behavior occurs among the newborn spiders, in which the mother may sometimes participate. This cannibalism ensures that only the strongest individuals survive, as noted by Hayyolalam and Kazem [6].

#### 3.4.2. Mathematical Modeling of *BWO*

The *BWO* consists of five steps:

- Initialization.
- Procreation.
- Cannibalism.
- Mutation.
- Termination criteria.

***Initialization***

As with other population-based metaheuristic algorithms, the optimization process starts with the generation of an initial population. In the *BWO* framework, each individual represents a candidate solution, referred to as a Widow, defined as $Widow=[w_{1},w_{2},\ldots ,w_{N_{var}}]$, where $N_{var}$ denotes the number of decision variables of the optimization problem. Each Widow belongs to the population *W*, and its decision variables $\left(w_{1},w_{2},\ldots ,w_{N_{var}}\right)$ are initialized using continuous random values that satisfy the problem constraints.

As a result, the initial population can be represented as a matrix of dimensions $N_{pop}\times N_{var}$, where $N_{pop}$ corresponds to the total number of Widows. The structure of the initial population matrix is presented in Equation (11).

(11) $$W=\begin{array}{cc} Widows_{1} & =\\ Widows_{2} & =\\ \vdots & \\ Widows_{N_{pop}} & = \end{array}\left[\begin{array}{ccccc} w_{1,1} & w_{1,2} & w_{1,3} & \cdots & w_{1,N_{var}}\\ w_{2,1} & w_{2,2} & w_{2,3} & \cdots & w_{2,N_{var}}\\ \vdots & \vdots & \vdots & \ddots & \vdots \\ w_{N_{pop},1} & w_{N_{pop},2} & w_{N_{pop},3} & \cdots & w_{N_{pop},N_{var}} \end{array}\right]$$

***Procreate***

In this phase, pairs of parent solutions are randomly selected to take part in the procreation mechanism. In the natural behavior of black widows, a large number of offspring is produced, although only the strongest individuals survive. To emulate this process, a randomly generated array denoted by α is introduced, which is used to produce new offspring solutions according to the following formulation:

(12) $$\left\{\begin{array}{c} CH_{1}=\alpha \times Par_{1}+\left(1-\alpha \right)\times Par_{2}\\ CH_{2}=\alpha \times Par_{2}+\left(1-\alpha \right)\times Par_{1} \end{array}\right.$$

In Equation (12), $Par_{1}$ and $Par_{2}$ denote the selected parent solutions, while $CH_{1}$ and $CH_{2}$ represent the corresponding offspring. This operation is repeated $N_{var}/2$ times, ensuring that $Par_{1}$ and $Par_{2}$ are distinct. Subsequently, both parents and offspring are subjected to the cannibalization process, and the surviving individuals are saved in a population matrix and ranked according to their cost values.

***Cannibalism***

In the *BWO*, the selection mechanism is inspired by three forms of cannibalistic behavior observed in nature, which are described as follows:

- **Sexual Cannibalism:** In line with the behavior in which a female black widow consumes the male during or after mating, the algorithm assigns gender roles according to fitness values, where the individual with the better fitness is designated as the female.
- **Sibling Cannibalism:** Similar to the natural process in which stronger offspring eliminate weaker siblings, the algorithm determines the number of surviving individuals based on the Cannibalism Rate (*CR*). Fitness values are used to differentiate between stronger and weaker black widows.
- **Maternal Cannibalism:** This mechanism reflects the situation in which offspring with better fitness eliminate their mother, allowing only the strongest individuals to remain in the population.

***Mutation Process***

**Figure 2:**
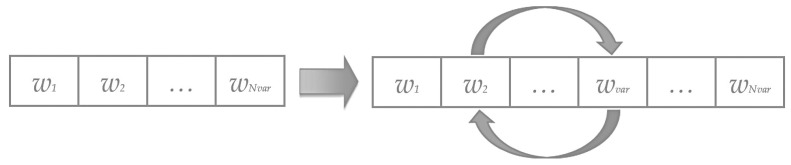
Mutation mechanism.

The mutation procedure starts by selecting nm individuals from the population obtained after the procreation stage, where the value of nm is defined by the Mutation Rate (*PM*). The Widows in this subset undergo a positional swap between two of their decision variables (as illustrated in Figure 2), resulting in a new candidate solution.

***Termination Criteria***

The definition of appropriate stopping conditions is a key aspect of population-based metaheuristic algorithms. In the proposed approach, three termination criteria are considered: (1) reaching a predefined maximum number of iterations, (2) when no improvement is observed in the best fitness value over a specified number of iterations, and (3) achieving a predefined fitness level.

From an optimization perspective, the biomimetic principles supporting *BWO* translate into well-defined search operators. Procreation and mutation mainly contribute to exploration by generating diversified candidate solutions, while cannibalism acts as a strong selection and intensification mechanism by systematically eliminating the weakest individuals. In optimization terms, this cannibalistic behavior functions as an aggressive selection operator that accelerates convergence by retaining only high-quality solutions. Within the context of the GCFP-MR, this selection pressure favors solutions that exhibit coherent machine–cell assignments, feasible routing decisions, and reduced intercellular movements under reliability constraints. The interaction of these mechanisms results in a dynamic equilibrium between exploration and exploitation, which is explicitly reflected in the pseudocode structure presented in Algorithm 1.
**Algorithm 1** Black Widow Optimization Algorithm.  1:**Input:** $Max_{iter}$, $N_{Pop}$, *Procreating Rate (PR)*, *Cannibalism Rate (CR)* and *Mutation Rate (PM)*  2:**Output:** The updated population $W^{\prime }=\{Widow_{1}^{\prime },Widow_{2}^{\prime },\ldots ,Widow_{N_{pop}}^{\prime }\}$ and Best  3:Initialize population *W*  4:**repeat**  5:    Determine number of reproduction pairs *# Procreation and cannibalism*  6:    **for** h=1 to nr **do**  7:        Choose two widows randomly $\left(Par_{1},Par_{2}\right)$  8:        Generate $N_{var}$ children using Equation (12)  9:        Destroy father10:        Eliminate offspring based on Procreation Rate (*PR*)11:        Save surviving solutions in $pop_{2}$12:    **end for**13:    Determine number of mutations nm=nr×PM *# Mutation*14:    **for** h=1 to nm **do**15:        Select a solution from $pop_{1}$16:        Mutate one decision variable randomly17:        Save mutated solutions into $pop_{3}$18:    **end for**19:    Update $W=pop_{2}+pop_{3}$20:**until** stopping criterion21:**return** best widow from *W*

**Figure 3:**
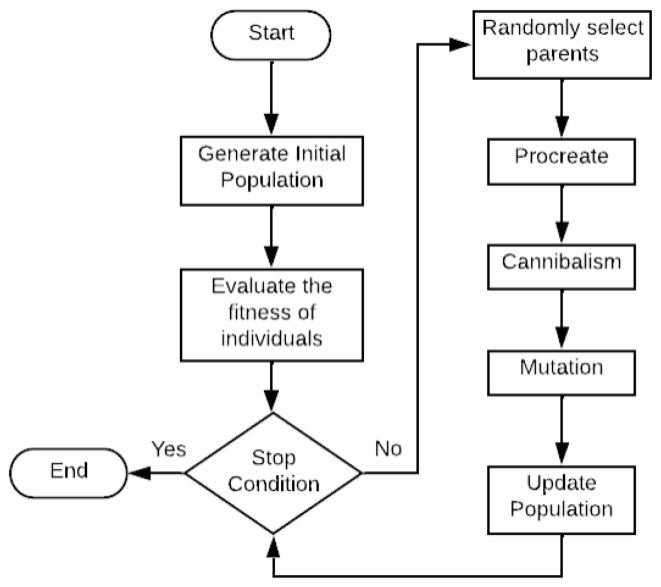
*BWO* diagram.

To further facilitate the understanding of the sequence of operations involved in the execution of the *BWO*, a schematic representation of the method is provided in Figure 3.

### 3.5. Golden Eagle Optimizer

#### 3.5.1. Inspiration: Golden Eagles’ Hunting Strategy

The *GEO* is inspired by the hunting behavior of the golden eagle, a bird of prey known for its exceptional vision and predatory skills. During the hunting process, these eagles exhibit a balanced transition between two fundamental phases: search and attack. The eagle systematically patrols its territory, scanning for potential prey while maintaining a spiral flight path that allows it to cover large areas efficiently. Once a target is identified, the eagle adjusts its flight trajectory, shifting from a broad search to a focused descent or attack phase. This behavioral duality, in which the eagle must choose between flying high to discover new hunting grounds or diving down to catch located prey, provides a robust framework for navigation through complex search spaces, as described by Mohammadi-Balani et al. [7].

#### 3.5.2. Mathematical Modeling of *GEO*

The *GEO* consists of five steps:

- Initialization.
- Parameter adjustment.
- Prey selection and movement.
- Memory update.
- Termination criteria.

***Initialization***

The optimization process in *GEO* begins with an initial population. Each individual is represented as the position of a golden eagle defined by the vector $Eagle=[e_{1},e_{2},\ldots ,e_{N_{var}}]$, where $N_{var}$ denotes the number of decision variables. These variables are initialized with random continuous values within the feasible search space defined by the problem constraints. Consequently, the entire population *E* is structured as a matrix, called the eagle’s memory, of dimensions $N_{pop}\times N_{var}$, where $N_{pop}$ represents the total number of eagles. The structure of this initial population matrix is presented in Equation (13).

(13) $$E=\begin{array}{cc} Eagle_{1} & =\\ Eagle_{2} & =\\ \vdots & \\ Eagle_{N_{pop}} & = \end{array}\left[\begin{array}{ccccc} e_{1,1} & e_{1,2} & e_{1,3} & \cdots & e_{1,N_{var}}\\ e_{2,1} & e_{2,2} & e_{2,3} & \cdots & e_{2,N_{var}}\\ \vdots & \vdots & \vdots & \ddots & \vdots \\ e_{N_{pop},1} & e_{N_{pop},2} & e_{N_{pop},3} & \cdots & e_{N_{pop},N_{var}} \end{array}\right]$$

***Parameter adjustment***

The coefficients that control the balance between exploration and exploitation are the attack propensity $p_{att}$ and the cruise propensity $p_{cru}$, which are set to initially favor exploration and gradually transition toward exploitation as iterations progress, where *t* is the current iteration and *T* is the maximum number of iterations.

(14) $$\left\{\begin{array}{c} p_{att}=p_{att}^{0}+\frac{t}{T}\left(p_{att}^{T}-p_{att}^{0}\right)\\ p_{cru}=p_{cru}^{0}-\frac{t}{T}\left(p_{cru}^{T}-p_{cru}^{0}\right) \end{array}\right.$$

***Prey selection and movement***

Each eagle selects a prey from the population memory and moves toward it using two vectors: the attack vector $\vec{ATT_{h}}$, for eagle *h*, which directs the eagle toward its prey (exploitation), and the cruise vector, which explores the area around it (exploration). To calculate the attack vector, Equation (15) is presented.

(15) $${\vec{ATT}}_{h}=\vec{E_{f}^{*}}-{\vec{E}}_{h}$$

The $\vec{E_{f}^{*}}$ term represents the best position found so far by the eagle, that is, the prey’s location saved in the memory, while ${\vec{E}}_{h}$ denotes the current position of the *h*-th eagle. This vector guides the individual’s movement directly toward the target to refine the solution. On the other hand, the cruise vector, which explores nearby solutions, is presented in Equation (16).

(16) $${\vec{CRU}}_{h}=(cru_{1}=\mathrm{random},\ldots ,cru_{v}=\frac{d-\sum_{u\neq v}att_{u}\;e_{u}}{att_{v}},\ldots ,cru_{Nvar}=\mathrm{random})$$

The destination point ${\vec{CRU}}_{h}$ on the cruise hyperplane is determined by identifying its *v*-th element, $cru_{v}$. This calculation involves the *u*-th element of the attack vector ${\vec{ATT}}_{h}$, denoted as $att_{u}$, and the specific element $att_{v}$ corresponding to the index of the fixed variable. To ensure that the algorithm places the resulting destination point randomly but systematically within the cruise hyperplane, it uses the scalar value *d* from Equation (17). This mechanism allows the golden eagle to maintain its trajectory relative to the prey while exploring alternative paths in the search space.

(17) $$d=\sum_{u=1}^{N_{var}}att_{u}\;e_{u}^{*}$$

The transition of the golden eagle’s position is governed by a step vector $\Delta e_{h}$ that integrates both hunting strategies. This vector represents the combined influence of the attack and cruise components, allowing the algorithm to balance global exploration with local intensification. The step vector is defined in Equation (18):

(18) $$\Delta e_{h}={\vec{r}}_{1}\;p_{att}\;\frac{{\vec{ATT}}_{h}}{∥{\vec{ATT}}_{h}∥}+{\vec{r}}_{2}\;p_{cru}\;\frac{{\vec{CRU}}_{h}}{∥{\vec{CRU}}_{h}∥}$$

In this formulation, $p_{att}$ and $p_{cru}$ are the attack and cruise coefficients, respectively, which regulate the priority given to each behavior. The terms ${\vec{r}}_{1}$ and ${\vec{r}}_{2}$ are random vectors with elements in the range [0,1], providing a stochastic nature to the movement. The normalized vectors $\frac{{\vec{ATT}}_{h}}{∥{\vec{ATT}}_{h}∥}$ and $\frac{{\vec{CRU}}_{h}}{∥{\vec{CRU}}_{h}∥}$ ensure that the direction of the movement is maintained regardless of the magnitude of the individual components. Finally, the new position of the *h*-th eagle for the subsequent iteration t+1 is updated by adding the calculated step vector to its current coordinates, as expressed in Equation (19):

(19) $$e_{h}^{t+1}=e_{h}^{t}+\Delta e_{h}^{t}$$

***Memory update***

The *GEO* maintains a memory of the best positions found by each eagle during the search process. After each position update, the continuous values of the new location are discretized before fitness evaluation. This is done through a simple rounding procedure, where each value is assigned to the nearest feasible integer associated with its corresponding route or cell option. If the new position yields a better fitness value than the one stored in the eagle’s memory, the memory is updated with the new coordinates. This memory-driven approach ensures that the population progressively converges toward the global optimum by using the best-known locations as targets for future attack vectors. If the new position $e_{h}^{t+1}$ improves the previous best position stored in the eagle’s memory, the memory is updated accordingly:

(20) $$\mathrm{if}\;Fitness^{t+1}<Fitness^{*}\;\Rightarrow \;Fitness^{*}=Fitness^{t+1}$$

***Termination criteria***

The algorithm iterates through these stages until the maximum number of iterations *T* or another stopping condition is met, after which the best solution found is reported. From an optimization perspective, the biomimetic principles that support the *GEO* are translated into a structured navigation strategy over the search space.

The cruise and attack behaviors jointly regulate the balance between exploration and exploitation, where the cruise phase enables wide-area search through spiral movements, while the attack phase progressively intensifies the search toward high-quality regions. In the context of the GCFP-MR, this mechanism facilitates an effective traversal of the large and highly constrained combinatorial space, allowing the algorithm to explore alternative machine–cell configurations and routing decisions without becoming prematurely trapped in local optima. The continuous interaction between cruise and attack dynamics ensures a coherent search process, which is explicitly reflected in the algorithmic structure summarized in Algorithm 2.

**Figure 4:**
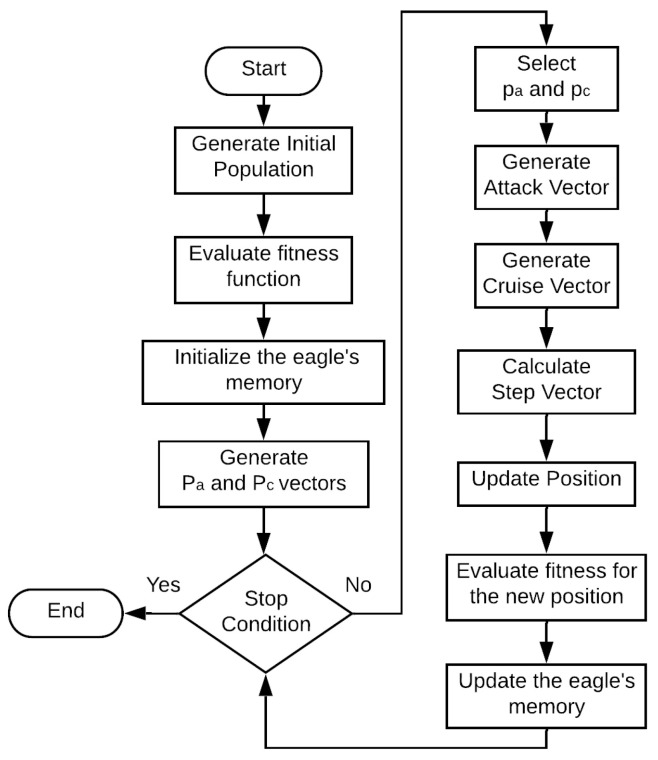
*GEO* diagram.

To facilitate understanding of all the steps necessary to correctly execute the *GEO*, Figure 4 is presented.
**Algorithm 2** Golden Eagle Optimizer Algorithm.  1:**Input:** $Max_{iter}$, $N_{Pop}$, *Attack propensity range* $\left(p_{att}^{0},p_{att}^{T}\right)$ and *Cruise propensity range* $\left(p_{cru}^{0},p_{cru}^{T}\right)$  2:**Output:** Final population $E^{\prime }=\left\{E_{1}^{\prime },E_{2}^{\prime },\ldots ,E_{N_{Pop}}^{\prime }\right\}$ and Best  3:Initialize population *E* randomly  4:Evaluate fitness for each $E_{h}$  5:Initialize memory with best position of each eagle  6:**repeat**  7:    Update $p_{att}$ and $p_{cru}$ according to iteration (Equation (14)) *# Transition to exploitation*  8:    **for** h=1 to $N_{Pop}$ **do**  9:        Randomly select prey *f* from memory10:        Compute attack vector ${\vec{ATT}}_{h}=E_{f}^{*}-E_{h}$ *# Exploitation*11:        Construct cruise hyperplane and generate cruise vector ${\vec{CRU}}_{i}$ *# Exploration*12:        Compute step $\Delta E_{h}=r_{1}\;p_{att}\;\frac{{\vec{ATT}}_{h}}{∥{\vec{ATT}}_{h}∥}+r_{2}\;p_{cru}\;\frac{{\vec{CRU}}_{h}}{∥{\vec{CRU}}_{h}∥}$ (Equation (18))13:        Update position $E_{h}=E_{h}+\Delta E_{h}$14:        Evaluate fitness of $E_{h}$15:        **if** fitness ($E_{h}$) better than memory (*h*) **then**16:           Update memory (*h*) = $E_{h}$17:        **end if**18:    **end for**19:**until** maximum iterations or convergence criterion20:**return** best solution from memory

### 3.6. Proposed Hybrid Strategies

The *BWO* stands out for its ability to intensify the search around promising regions through its mechanisms of procreation, cannibalism, and mutation, making it very effective in the exploitation phase [6]. In contrast, the *GEO* offers a robust balance between exploration and exploitation thanks to its dynamic strategy of transitioning from a “cruise” to an “attack” behavior, allowing it to maintain diversity and ensure stable convergence [7].

According to Blum et al. [26], metaheuristics research for combinatorial optimization has experienced a significant transition toward hybridization strategies. At the same time, the emphasis has shifted from focusing predominantly on algorithms to focusing more on problems, as in the case of GCFP-MR.

From a structural and algorithmic design perspective, *GEO* is particularly well-suited to act as the core of a hybrid framework due to its closely linked exploration–exploitation mechanism. In *GEO*, the cruise and attack phases are intrinsically linked, as the cruise vector depends directly on the attack vector, forming a coherent and continuous search dynamic. This integrated behavior provides a stable and coherent search process. In contrast, *BWO* operators, such as cannibalism, mutation, and selection, are modular and can be applied independently. This modularity makes *BWO* particularly well-suited as a source of intensification mechanisms that can be integrated into the *GEO* structure without altering its fundamental search logic. Therefore, the hybridization strategy is justified by the complementary role of both algorithms: *GEO* preserves a coherent balance between exploration and exploitation, while selected *BWO* operators introduce additional perturbation and selection mechanisms that can help the search escape local optima and refine promising solutions without disrupting the core *GEO* dynamics.

Based on the above considerations, three hybrid strategies are proposed in this study. The first strategy, denoted as Golden Eagle Optimizer and Black Widow Optimizer Hybrid Algorithm ($GEO-BWO^{HA}$), applies both metaheuristics in their complete form, where the population is sequentially processed by *GEO* and subsequently by *BWO* at each iteration, thus combining the global search capability of *GEO* with the intensification mechanisms of *BWO*. The second strategy, referred to as Golden Eagle Optimizer with the Procreation and Cannibalism from the BlackWidow Optimizer ($GEO^{PR-CR}$), represents a partial hybridization in which the procreation and cannibalism operators of *BWO* are embedded within the *GEO* framework in order to strengthen diversification and selective pressure while preserving the inherent exploration–exploitation balance of *GEO*. Finally, the third strategy, denoted as Golden Eagle Optimizer with Mutation from the Black Widow Optimizer (*GEO^PM^*), incorporates the mutation operator of *BWO* into the *GEO* search process with the aim of enhancing exploratory behavior, mitigating premature convergence, and promoting a broader exploration of the solution space.

**Figure 5:**
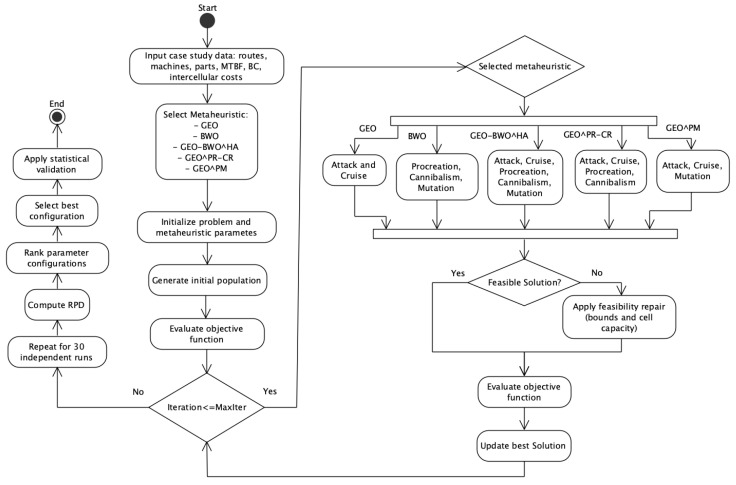
Proposed methodological process for evaluating reference and hybrid metaheuristics applied to the GCFP-MR.

To provide a clearer overview of the proposed procedure, Figure 5 presents a detailed flowchart of the methodological process followed in this study. The diagram summarizes how the metaheuristics are evaluated, starting from the initialization of the case study and algorithm parameters, followed by the selection of the corresponding strategy, the application of the search operators, feasibility verification, solution repair when required, objective function evaluation, and final performance comparison.

## 4. Results and Discussion

All computational experiments were implemented in Python 3.13.2 and executed on two computers: one equipped with an Intel Core i7 14th-generation processor and 16 GB of RAM, and another equipped with an Apple M1 processor and 8 GB of RAM. The algorithms were evaluated through independent stochastic runs using randomly generated initial populations. A fixed random seed was not imposed in the original experimental campaign, since the purpose was to assess the average behavior of the metaheuristics under independent random initializations. Although this prevents exact bitwise replication of the random sequences, the complete experimental configuration, including parameter settings, number of runs, stopping criteria, and statistical analysis procedures, is reported to support methodological reproducibility. The source code and experimental data are available from the corresponding author upon reasonable request.

It should be noted that this study uses discrete metaheuristics rather than binary encodings. As highlighted by Sawaya et al. [42] and Tamura et al. [43], discrete representations typically involve integer or natural numbers, while binary representations use values {0, 1} to indicate dichotomous decisions. The difference lies in how solutions are represented during the solving process. Binary coding requires expanding each alternative into multiple positions. For example, if part A has to choose between three routes, choosing route 3 would be represented as [0,0,1], and for part B the choice is route 2, it would be coded as [0,1,0]. This representation results in long binary data vectors, increases redundancy, and forces the algorithm to apply additional feasibility checks and repair mechanisms after genetic operations such as crossover or mutation (*BWO*) and hunting operations such as cruise and attack (*GEO*), which increases the computational time required [44].

In contrast, a discrete representation allows each alternative to be expressed directly with integer values. In the same example, selecting route 3 for part A and route 2 for part B can be written simply as the vector [3,2], where each position in the data vector denotes the route chosen for each part. This encoding is more compact, avoids unnecessary dimensionality, and naturally reflects the structure of the problem. Furthermore, it allows for the design of more intuitive and problem-oriented search operators, such as switching machines between cells or reassigning a part to another route. These operators can be implemented directly on integer vectors in Python, without the need for binarization or unnecessary additional repair procedures [45].

Therefore, adopting a discrete representation improves the efficiency of the search, reduces computational overhead, and provides a more intuitive exploration of feasible solutions. This approach allows metaheuristics such as *GEO* and *BWO* to use their exploratory capabilities when solving the CFP with additional factors, such as alternative routes and reliability, in a more effective way.

### 4.1. Relative Percentage Deviation

Algorithms such as *GEO* and *BWO* may produce different solutions across multiple executions, even when applied under identical conditions. To ensure a fair comparison between results and evaluate the consistency of both methods, the Relative Percentage Deviation (RPD) is commonly used, as it provides a standardized criterion for comparing the performance of different algorithms [46].

According to Lanza-Gutierrez et al. [47], the RPD measures how far a given solution deviates from the best or optimal value found, providing a normalized indicator of performance. It is calculated as follows:

(21) $$RPD=\frac{{\bar{Cost}}_{comb}-Cost_{opt}}{Cost_{opt}}\cdot 100$$

where ${\bar{Cost}}_{comb}$ denotes the average cost obtained for a specific configuration, and $Cost_{opt}$ is the best fitness achieved among all runs.

### 4.2. Solution Representation

In the implementation of metaheuristics for the GCFP-MR, solution vectors are generated to satisfy the constraints defined in Section 3.1, with a size of *Nvar* (17 in this case). This dimension corresponds to eight parts, each with the possibility of selecting a specific route, and nine machines that can be distributed among the cells. Each element of the solution vector is encoded as a positive integer that, depending on its position, represents either the route assigned to a part (the first n=8 elements) or the manufacturing cell to which a machine is allocated (the remaining m=9 elements).

Following the work of Figueroa-Torrez et al. [25], in which the solution to the GCFP-MR was represented by a binary-encoded vector, the same solution is represented in this study through a discrete vector. In contrast, our formulation employs an integer encoding within a discrete model, as presented below.

$$Vector^{BS}=\left[1,2,2,1,2,2,1,1,2,1,1,2,2,2,2,1,2\right]$$

The $Vector^{BS}$ represents the best solution obtained and yields a total cost of USD $4671.34. This total and optimal cost was calculated using an Exhaustive Search (ES) based on the route and MTBF data presented in Table 2 and Table 3. These tables were originally proposed by Bhide et al. [48], and were subsequently adapted slightly by Jouzdani et al. [33] and Alhourani [49]. Table 2 illustrates the available routes for each part and the costs associated with each intercellular movement in each route. Additionally, it can be observed that this table shows the machines required for the production of each part and their corresponding processing times, expressed in minutes. Finally, Table 3 presents MTBF and Breakdown Cost (BC) for each machine.

Due to the stochastic nature of metaheuristic algorithms, each configuration was evaluated over multiple independent runs. Following common practice in the metaheuristics literature, 30 runs were performed to obtain statistically stable average results [25,50,51].

### 4.3. Solution Repair Process

During the execution of metaheuristic operators, it is possible to generate infeasible solution vectors that do not satisfy one or more constraints of the GCFP-MR. In highly constrained optimization problems, discarding such solutions may significantly reduce search efficiency and slow down convergence toward high-quality feasible regions. For this reason, repair-based mechanisms are commonly employed to transform promising infeasible solutions into feasible ones, allowing the algorithm to exploit valuable search information while maintaining feasibility throughout the optimization process [52,53].

Recent studies have incorporated repair processes to address similar feasibility issues. For example, Crawford et al. [54] applied a repair mechanism in a binary adaptation of the Dream Optimization Algorithm (DOA) for the Set Covering Problem (SCP), enabling the metaheuristic to restore infeasible solutions generated during the search and operate effectively in a highly constrained binary optimization domain.

For this reason, a solution repair mechanism was implemented to ensure that each generated solution complies with the basic feasibility requirements of the mathematical model. After each solution is modified by an exploration or exploitation operator, a feasibility verification step is first performed. If the solution satisfies the feasibility conditions defined by the structural constraints of the model, it is passed directly to the objective function evaluation. Otherwise, the repair mechanism is called before fitness evaluation, ensuring that only feasible solution vectors are evaluated and propagated to the next population.

The repair mechanism takes an infeasible solution vector and modifies it to satisfy all structural restrictions: route assignment per part (Equation (2)), cell assignment per machine (Equation (3)), and the upper and lower limits on the number of machines per cell (Equations (4) and (5)). The repair process acts sequentially through two main correction phases and one global feasibility verification loop, described as follows.

- *Phase 1: Correction of individual bounds.*
  The first step verifies that each element of the solution vector is within its feasible domain. The vector is composed of two segments: the first *n* elements correspond to the routes selected for each part, while the remaining *m* elements correspond to the cell assigned to each machine. For the route-selection variables associated with each part *i*, the upper bound is given by $q_{i}$, which specifies the number of routes for that part *i*. For the machine–cell assignment variables, the upper bound corresponds to the total number of cells *c*. For each position *i* of the vector, if the current value is infeasible, it is replaced by a random feasible value within the corresponding interval ($routes\in \{1,\ldots ,q_{i}\}$ and machines∈{1,…,c}). This ensures that all route and machine indices belong to their admissible sets, restoring the domain consistency of the solution vector before any further checks.
- *Phase 2: Distribution of machines across cells.*
  After individual bounds are corrected in *Phase 1*, the repair mechanism proceeds to validate the cell capacity constraints (Equations (4) and (5)), defined by the upper and lower bounds ($U_{l}$ and $L_{l}$) for each cell l∈{1,…,c}. The algorithm builds a temporary vector containing only the last *m* elements, which represent the cell assignments of all machines. Then, the number of machines per cell is computed using a frequency counter. If any cell does not satisfy its capacity constraints, the algorithm randomly selects one machine index *k* and reassigns its cell value to a new random feasible cell. The process is repeated inside a loop until all cells satisfy the constraints. This stochastic reallocation maintains solution diversity while gradually restoring global feasibility regarding machine distribution among cells.

The two previous phases are reevaluated iteratively until every constraint is satisfied and the solution becomes fully feasible. This is efficiently achieved by maintaining the “while” control variable, which only terminates when all cells simultaneously meet the lower and upper capacity bounds. In practice, feasibility is guaranteed as long as the condition is satisfied.

In the implemented procedure, mutation is performed as a positional swap between two randomly selected elements of the solution vector. Therefore, its effect depends on the segment to which the selected positions belong. If one selected position belongs to the route segment and the other belongs to the cell segment, the swap may generate an infeasible solution due to the different numerical domains associated with route and cell values. Similarly, a swap between two route positions may become infeasible when the transferred route value exceeds the number of available routes for the corresponding part. For this reason, after each mutation, the resulting vector is checked for feasibility before the objective function is evaluated. If the mutated solution violates the structural constraints of the model, the repair mechanism is called to restore feasibility before fitness evaluation. This same feasibility verification and repair sequence is applied after all exploration and exploitation operators, regardless of the selected metaheuristic. This repair procedure effectively prevents the propagation of infeasible individuals in the population, maintaining the validity of candidate solutions throughout the evolutionary process.

### 4.4. GCFP-MR in BWO

In order to determine the optimal configuration of the *BWO* algorithm applied to the GCFP-MR, an exhaustive search was conducted on the solution vector presented in Section 4.2, evaluating multiple combinations of population size, maximum number of iterations, and the key algorithmic parameters: Procreation Rate (*PR*), *CR*, and *PM*.

The sets of values considered are as follows:

- Population size ∈ {25, 50, 75, 100},
- Maximum iterations ∈ {25, 50, 75, 100},
- PR ∈ {0.2, 0.4, 0.6, 0.8},
- CR ∈ {0.2, 0.4, 0.6, 0.8},
- PM ∈ {0.2, 0.4, 0.6, 0.8}.

The combination of these sets resulted in a total of 1024 distinct configurations (4×4×4×4×4), each evaluated using RPD as the performance metric.

The best parameter configuration was obtained with:

- $N_{\mathrm{pop}}=100,$
- Iter=50,
- PR=0.8,
- CR=0.8,
- PM=0.8

This configuration achieved an RPD of **0.855%**, the lowest among all configurations evaluated for this algorithm, thereby demonstrating its superior effectiveness in solving the GCFP-MR within the analyzed search space. The best ten RPD values are presented in Table 4. These top 10 positions were obtained from 30,720 experiments, as each of the 1024 combinations was evaluated 30 times.

Having established the optimal configuration of the *BWO* and its results in the GCFP-MR, the following subsection introduces the implementation of the *GEO* to contrast its performance with that of the previously analyzed algorithm.

### 4.5. GCFP-MR in GEO

The *GEO* is applied to the GCFP-MR with the aim of evaluating its exploration and exploitation capabilities within the search space. Similar to the procedure followed with the *BWO*, an exhaustive parameter search was performed to identify the optimal configuration of the algorithm. In order to determine the optimal setup of *GEO*, multiple combinations of population size, maximum number of iterations, and initial–final ranges of the attack ($p_{a}$) and cruise ($p_{c}$) propensities were evaluated. Specifically, the following parameter sets were considered:

- Population size ∈ {25, 50, 75, 100}.
- Maximum iterations ∈ {25, 50, 75, 100}.
- $p_{a}$∈ {[0.0, 0.5], [0.0, 1.0], [0.0, 1.5], [0.0, 2.0], [0.5, 1.0]
    [0.5, 1.5], [0.5, 2.0], [1.0, 1.5], [1.0, 2.0], [1.5, 2.0]}.
- $p_{c}$∈ {[1.00, 0.75], [1.00, 0.50], [1.00, 0.25], [1.00, 0.00], [0.75, 0.50]
    [0.75, 0.25], [0.75,0.00], [0.50, 0.25], [0.50, 0.00], [0.25, 0.00]}.

The combination of these sets resulted in a total of 1600 distinct configurations (4×4×10×10), each of which was evaluated using the RPD as the performance metric.

The results indicate that the best parameter configuration corresponds to:

- $N_{\mathrm{pop}}=100$.
- Iter=100.
- $p_{a}=\left[1.5,\;2.0\right]$.
- $p_{c}=\left[1.00,\;0.25\right]$.

This configuration achieved an RPD of **1.068%**, the lowest among all combinations tested, thereby demonstrating the best performance of *GEO* in solving the GCFP-MR for the evaluated set of instances. The best ten RPD values are presented in Table 5. These top 10 positions were obtained from 48,000 experiments, as each of the 1600 combinations was evaluated 30 times.

Building on the insights gained from both the *BWO* and *GEO* individually, the following section explores their hybridization, aiming to combine the strengths of both metaheuristics in order to further enhance solution quality.

### 4.6. Hybridization 1: GCFP-MR in GEO-BWO^HA^

To evaluate the behavior of the complete hybrid scheme, an exhaustive search of parameter combinations was conducted to identify the optimal configuration of the $GEO-BWO^{HA}$ algorithm. The tested parameters included population size, maximum number of iterations, the attack and cruise propensities of *GEO* ($p_{a}$, $p_{c}$), and the reproduction, cannibalism, and mutation rates of *BWO* (*PR*, *CR*, *PM*). Taking into account the parameter sets defined in the previous sections, the combination of these values generated a total of **102,400 distinct configurations** (4×4×10×10×4×4×4). Considering that each configuration was independently executed 30 times to ensure statistical robustness, this corresponds to a dataset comprising **3,072,000 experiments** (102,400×30), each evaluated using the RPD as the performance metric. The results indicate that the best performance was achieved with the following configuration:

- $N_{\mathrm{pop}}=100$.
- Iter=50.
- $p_{a}=\left[0.5,\;1.5\right]$.
- $p_{c}=\left[1.00,\;0.50\right]$.
- PR=0.8.
- CR=0.4.
- PM=0.8.

This configuration yielded an RPD of **0.734%**, the lowest among all the tested settings, thereby demonstrating the effectiveness of the $GEO-BWO^{HA}$ approach in solving the GCFP-MR. The best ten RPD values are presented in Table 6. These top 10 positions were obtained from the 3,072,000 experiments mentioned above.

### 4.7. Hybridization 2: GCFP-MR in GEO^PR−CR^

In order to assess the impact of incorporating selective and reproductive mechanisms from *BWO* into the *GEO* framework, an exhaustive parameter search was performed for this hybrid scheme. The tested parameters included population size, maximum number of iterations, the attack and cruise propensities of *GEO* ($p_{a}$, $p_{c}$), and the procreation and cannibalism rates of *BWO* (*PR*, *CR*). The results indicate that the best parameter configuration was obtained with:

- $N_{\mathrm{pop}}=100$.
- Iter=50.
- $p_{a}=\left[1.5,\;2.0\right]$.
- $p_{c}=\left[0.50,\;0.25\right]$.
- PR=0.8.
- CR=0.6.

This configuration achieved an RPD of **1.162%**, which represents the best performance among all tested settings for this partial hybridization. The best ten RPD values are presented in Table 7. These top 10 positions were obtained from 768,000 experiments, as each of the 25,600 feasible combinations was evaluated 30 times.

### 4.8. Hybridization 3: GCFP-MR in ${\mathrm{GEO}}^{PM}$

To analyze the effect of introducing mutation as an exploratory mechanism within the *GEO* framework, an exhaustive search of parameter combinations was carried out. The tested parameters included population size, maximum number of iterations, the attack and cruise propensities of *GEO* ($p_{a}$, $p_{c}$), and the mutation rate *PM* of *BWO*. The combination of these parameter sets generated a total of 6400 distinct configurations (4×4×10×10×4), each evaluated using RPD as the performance metric. The best configuration obtained for this hybridization was:

- $N_{\mathrm{pop}}=100$.
- Iter=100.
- $p_{a}=\left[0.5,\;2.0\right]$.
- $p_{c}=\left[0.50,\;0.00\right]$.
- PM=0.8.

This configuration achieved an RPD of **0.592%**, which represents the lowest value among all 6,400 tested configurations, thus confirming the effectiveness of integrating the *BWO* mutation operator within the *GEO* search process. The best ten RPD values are presented in Table 8. These top 10 positions were obtained from 192,000 experiments, as each of the 6400 combinations mentioned above was evaluated 30 times.

### 4.9. Discussion and Analysis

The experiments reveal differences between the metaheuristics tested. The *GEO^PM^* variant achieves the lowest RPD (0.592%), outperforming all the alternatives in this study. Second place is achieved by $GEO-BWO^{HA}$ (0.734%). The independent *BWO* achieves 0.855%, while *GEO* achieves 1.068%, and the $GEO^{PR-CR}$ variant obtains an RPD of 1.162%.

These results suggest that *GEO^PM^* enhances the exploratory capacity of the algorithm, helping to maintain population diversity and prevent premature convergence to local optima within the discrete search space of the GCFP-MR. This improvement in exploration contributes to obtaining better solutions that are more accurate and have better RPD values than the previous solutions obtained by the other algorithms. Overall, the ranking by best RPD is:

$$GEO^{PM}\;\left(0.592\%\right)<GEO-BWO^{HA}\;\left(0.734\%\right)<BWO\;\left(0.855\%\right)<GEO\;\left(1.068\%\right)<GEO^{PR-CR}\;\left(1.162\%\right)$$

While these descriptive results highlight clear performance differences, a rigorous assessment is still required to determine whether such improvements are statistically significant across runs. Therefore, the following section applies a nonparametric validation using the Wilcoxon-Mann-Whitney (WMW) test to verify whether the lower RPD values obtained by *GEO^PM^* are statistically better than those produced by the other configurations.

## 5. Experimental Validation

To enhance the robustness of experimental results, it is essential to complement the performance evaluation with an appropriate statistical validation procedure. While raw performance metrics provide an initial indication of algorithmic behavior, they do not conclusively determine whether the observed differences in solution quality are statistically significant. Therefore, this section applies a nonparametric statistical test to rigorously assess whether the performance gaps among the compared algorithms reflect genuine differences rather than random variation.

### 5.1. Statistical Analysis: Wilcoxon–Mann–Whitney Test

Since the data come directly from the computational runs and do not exhibit normal behavior, statistical analysis must be performed using a nonparametric approach. Considering also that the results generated are independent, the most appropriate test is the WMW [55,56]. It should be mentioned that the WMW test is a nonparametric statistical method that allows two independent samples to be compared without requiring the assumption of normality [57]. It was implemented in Python using the *scipy* library (*scipy.stats.mannwhitneyu*). In this procedure, the “alternative” parameter is defined as “less”. Two different combinations ($Comb_{A}$ and $Comb_{B}$) are compared, and when the *p-value* is less than 0.05, it is established that sample $Comb_{A}$ is statistically smaller than sample $Comb_{B}$. Thus, the hypotheses are formulated as follows:

$$H_{0}:Comb_{A}\geq Comb_{B}$$

$$H_{1}:Comb_{A}<Comb_{B}$$

This indicates that if the resulting *p-value* is less than 0.05, the null hypothesis ($H_{0}$) is rejected, and it can be stated that $Comb_{A}$ obtains a statistically lower value than $Comb_{B}$. This interpretation confirms that the problem being analyzed is formulated as a minimization problem.

In GCFP-MR, where optimization techniques are applied to assign machines and parts to manufacturing cells efficiently, it is essential to evaluate the effectiveness of different metaheuristic approaches. The *GEO* and the *BWO* are the two algorithms evaluated in this research to rigorously determine whether one algorithm significantly outperforms the other in terms of solution quality. Analyzing the different RPD values obtained, it was observed that the hybridization of the *GEO^PM^* produced the best result. However, to determine whether this combination is truly superior, Table 9 presents the WMW comparison of the best-performing configurations of the algorithms introduced in Section 4: BWO, GEO, $GEO-BWO^{HA}$, $GEO^{PR-CR}$, and *GEO^PM^*.

After analyzing the WMW results in Table 9, it can be observed that there is no single algorithm that is statistically superior to all the others. Although $GEO^{PM}$ achieved the best descriptive performance, the WMW results indicate that it only presents statistically significant differences with respect to GEO and $GEO^{PR-CR}$. In contrast, no statistically significant differences were observed between $GEO^{PM}$, BWO, and $GEO-BWO^{HA}$. Therefore, the statistical evidence suggests the absence of a clear overall winner, leading instead to a competitive group composed of BWO, $GEO-BWO^{HA}$, and $GEO^{PM}$ in terms of solution quality.

An additional observation is that these three methods share a common mechanism: they incorporate the BWO mutation process. This recurrent pattern supports the hypothesis that the mutation component contributes substantially to performance. However, statistical non-significance alone does not explain how the algorithms behave across repeated runs, and does not clarify their practical precision and accuracy when executed multiple times. For this reason, a complementary graphical analysis is introduced in the next section to compare the distribution of final outcomes and the convergence behavior of the five algorithms.

### 5.2. Graphical Performance Analysis

This section presents a graphical comparison of the five evaluated algorithms (BWO, GEO, $GEO-BWO^{HA}$, $GEO^{PR-CR}$, and $GEO^{PM}$). For each algorithm, the parameter configuration associated with its best RPD was selected. In order to ensure a fair and instance-by-instance comparison, a set of 300 random initial populations was generated. Each initial population was then used as the starting point for all five algorithms, so that every method solved exactly the same 300 initial populations, producing 300 final fitness values per algorithm. Since the problem is formulated as a minimization problem, lower objective values indicate better performance. A box plot is first used to compare the distribution of final objective values across algorithms. Then, a convergence plot is provided for each algorithm to visualize the evolution of the best objective value at each iteration.

#### 5.2.1. Boxplot Comparison of Final Objective Values

**Figure 6:**
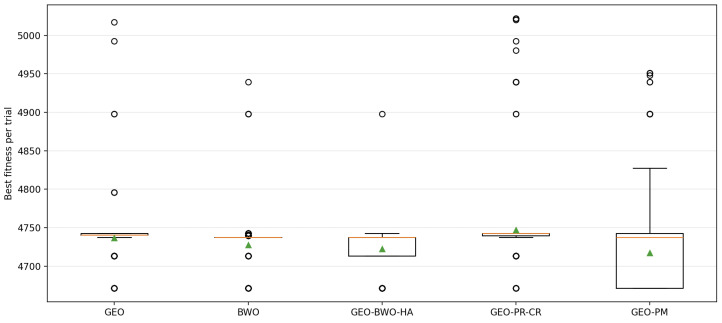
Boxplot with 300 runs for each algorithm.

Before discussing the results, it is convenient to briefly describe the elements of a box plot [58]. Each box summarizes the distribution of the final objective values obtained over the 300 shared initial populations. The box spans the interquartile range (IQR), from the first quartile ($Q_{1}$, 25th percentile) to the third quartile ($Q_{3}$, 75th percentile), and its height represents the dispersion of the central 50% of the observations. The horizontal line inside the box (orange) corresponds to the median (50th percentile). The whiskers extend toward the most extreme values that are not considered outliers, while points beyond the whiskers are plotted as outliers. Finally, the green triangle indicates the mean value across runs. In this minimization context, lower values imply better performance. Moreover, because the same set of initial populations is used for all algorithms, the box plot provides a direct indication of which method tends to solve the same initial populations more effectively. The boxplot is presented in Figure 6.

From a dispersion perspective, BWO exhibits the highest precision, as indicated by its relatively compact box (small IQR), meaning that the final results tend to be tightly concentrated. In contrast, when focusing on accuracy (i.e., proximity to the best-known/optimal value), the closest central tendencies are observed for $GEO^{PM}$ and $GEO-BWO^{HA}$, which can be interpreted as a practical tie in terms of how near their distributions lie to the optimal solution. However, $GEO^{PM}$ shows a slight advantage because it reaches the optimal solution more frequently (132 optimal solutions). Although $GEO-BWO^{HA}$ has a shorter whisker range, suggesting a more compact distribution, it reaches the optimum less frequently (69 optimal solutions). For this reason, BWO is highlighted as the most precise method, whereas $GEO^{PM}$ stands out as the most accurate in terms of closeness to the minimum objective value.

#### 5.2.2. Convergence Analysis

This subsection complements the boxplot analysis by examining the convergence behavior of the five evaluated algorithms. Since ${\mathrm{GEO}}^{PM}$ exhibited the most favorable distribution in the boxplots, the convergence figures provide further insight into whether this advantage is associated with faster improvement, greater stability, or a higher frequency of reaching the optimal value. For each algorithm, two figures are presented: one showing a focused view of the first 15 iterations, where the main convergence behavior can be more clearly observed, and a second one that displays additional enlarged views that allow for a better examination of the search results. Each curve represents the average value obtained from 300 random initial populations, all of which were solved using the best parameter configuration identified in Section 4 for the corresponding algorithm.

**Figure 7:**
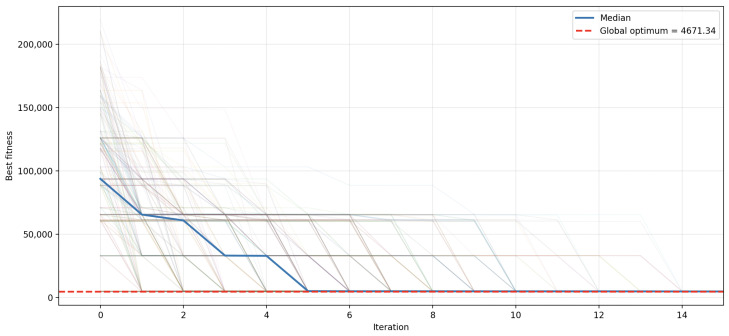
Focused convergence curve of *GEO* during the first 15 iterations.

**Figure 8:**
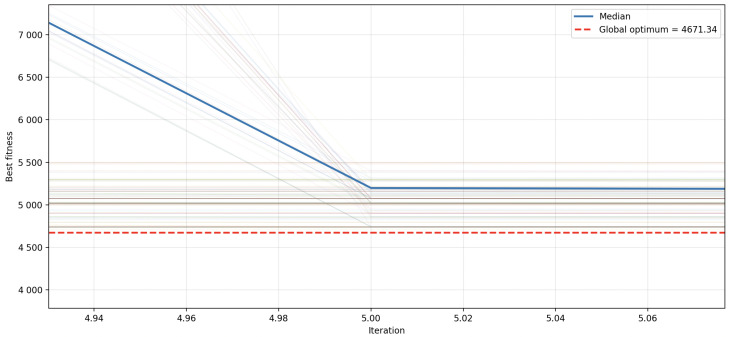
Zoomed view of the *GEO* convergence point. Faint colored lines represent individual runs.

For *GEO*, the algorithm was run with a configuration of 100 iterations. Its average convergence pattern shows relatively slower initial search behavior, with a steep initial descent occurring after approximately five iterations (Figure 7), at which point the average cost reaches about 5198 (Figure 8). Although *GEO* continues to improve beyond this stage, its ability to repeatedly reach the optimum remains limited, as the optimum value was only reached 19 times out of 300 runs. This suggests that, despite its ability to progressively refine solutions, its exploitation performance is weaker than that of the best-performing alternatives.

**Figure 9:**
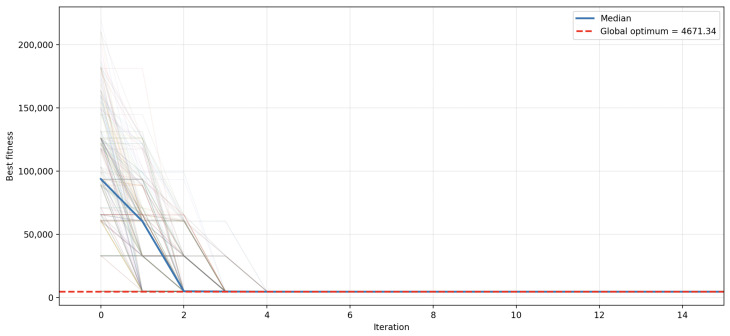
Focused convergence curve of *BWO* during the first 15 iterations.

**Figure 10:**
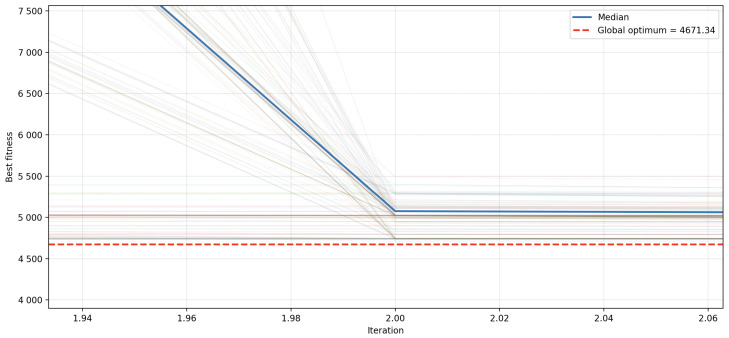
Zoomed view of the *BWO* convergence point. Faint colored lines represent individual runs.

In the case of *BWO*, the best configuration required 50 iterations. Its convergence pattern shows a much faster initial decline (Figure 9), which occurs after approximately two iterations and reaches an average cost of around 5075 (Figure 10). In addition, *BWO* reaches the optimal value 50 times, revealing a greater ability to repeatedly identify high-quality solutions. Compared to *GEO*, this behavior reflects a more effective balance between early intensification and solution refinement.

**Figure 11:**
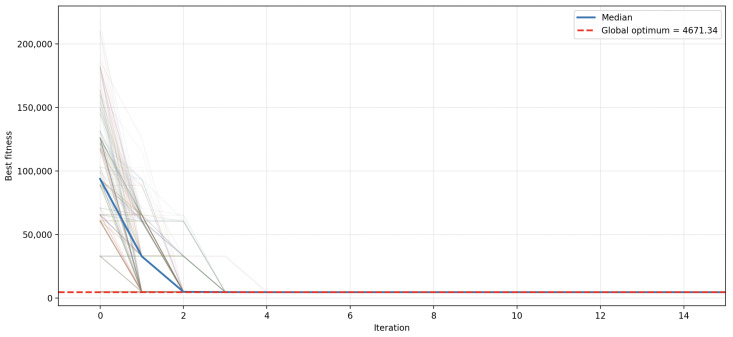
Focused convergence curve of ${\mathrm{GEO}-\mathrm{BWO}}^{HA}$ during the first 15 iterations.

**Figure 12:**
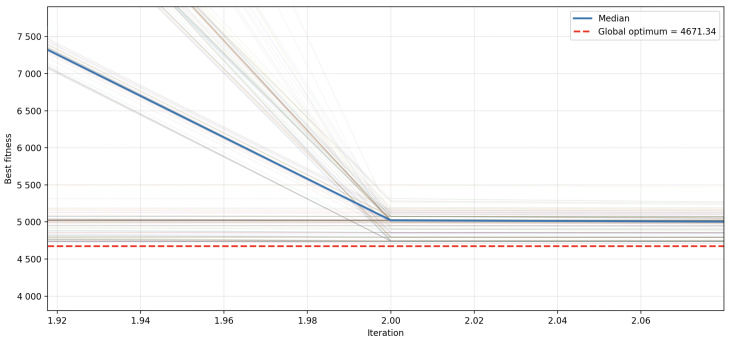
Zoomed view of the ${\mathrm{GEO}-\mathrm{BWO}}^{HA}$ convergence point. Faint colored lines represent individual runs.

The convergence behavior for $GEO-BWO^{HA}$ is also based on 50 iterations. As in *BWO*, its initial steep descent occurs after approximately two iterations (Figure 11), reaching an average cost close to 5022 (Figure 12). More importantly, this variant reaches the optimum 69 times, which represents an improvement over both individual parent algorithms.

**Figure 13:**
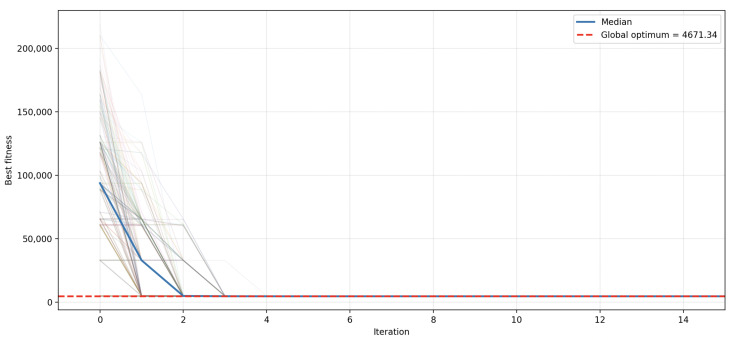
Focused convergence curve of ${\mathrm{GEO}}^{PR-CR}$ during the first 15 iterations.

**Figure 14:**
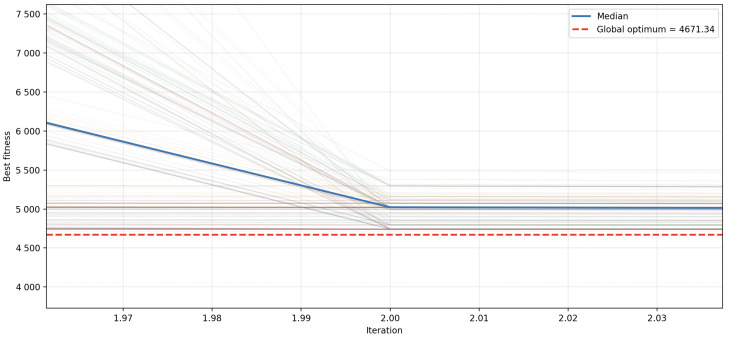
Zoomed view of the ${\mathrm{GEO}}^{PR-CR}$ convergence point. Faint colored lines represent individual runs.

The ${\mathrm{GEO}}^{PR-CR}$ variant also uses 50 iterations and exhibits early convergence behavior comparable to that of ${\mathrm{GEO}-\mathrm{BWO}}^{HA}$ (Figure 13), with a steep initial decline occurring after approximately two iterations and reaching an average cost close to 5022 (Figure 14). However, despite this similar initial trajectory, its frequency of reaching the optimum is considerably lower, with only 12 optimal results. This result indicates that showing a comparable average convergence level in the first iterations does not necessarily translate into a high capacity to consistently reach the optimal solution.

**Figure 15:**
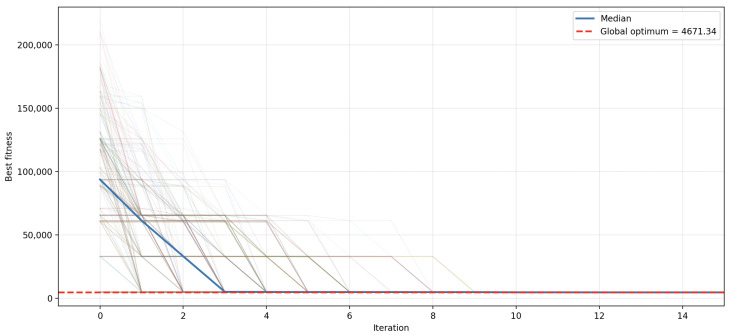
Focused convergence curve of ${\mathrm{GEO}}^{PM}$ during the first 15 iterations.

**Figure 16:**
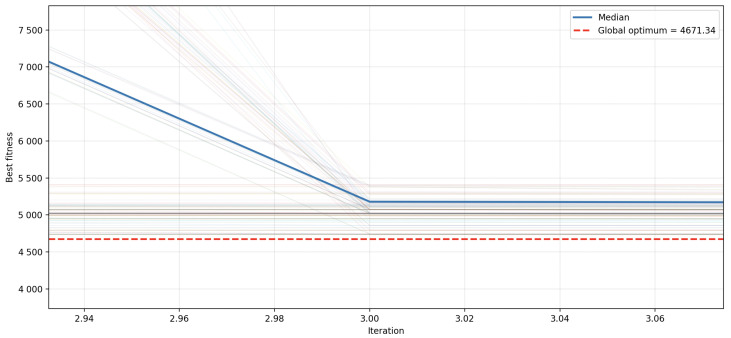
Zoomed view of the ${\mathrm{GEO}}^{PM}$ convergence point. Faint colored lines represent individual runs.

Finally, ${\mathrm{GEO}}^{PM}$ was executed with 100 iterations and shows a distinct behavior (Figure 15). Its curve reaches an early stabilization level within the first few iterations, approximately around the third iteration, with an average cost close to 5179 (Figure 16). Although this early average value is not the lowest among the evaluated methods, ${\mathrm{GEO}}^{PM}$ clearly stands out in terms of final effectiveness, since it attains the optimum 132 times. Therefore, its superiority appears to lie not in achieving the best average cost during the first convergence stage, but in maintaining a search dynamic that more consistently drives the process toward the optimal solution throughout the run.

A comparative analysis of the frequency of optimal hits further reinforces this conclusion. ${\mathrm{GEO}}^{PM}$ reaches the optimum 6.95 times more often than *GEO*, 2.64 times more often than *BWO*, 1.91 times more often than ${\mathrm{GEO}-\mathrm{BWO}}^{HA}$, and 11 times more often than ${\mathrm{GEO}}^{PR-CR}$. Consequently, the convergence analysis supports the evidence obtained from the boxplots: ${\mathrm{GEO}}^{PM}$ provides the most robust overall behavior among the evaluated methods. More importantly, these results suggest that performance improvements do not necessarily depend on combining two metaheuristics in their entirety. Instead, selectively incorporating an effective search operator, such as the mutation mechanism, may yield greater benefits than a complete hybridization structure.

### 5.3. Analysis of Computational Complexity with Big-O

The analysis of computational complexity, expressed through Big-O notation, is essential for understanding the scalability and efficiency of metaheuristic algorithms [59]. Big-O notation provides a mathematical framework to describe the worst-case time complexity of an algorithm, that is, how the number of operations increases with the size of the input [60].

In the context of the GCFP-MR, where the solution space grows exponentially with the number of parts and machines, this analysis offers valuable insights into the computational resources demanded by each metaheuristic. Accordingly, the Big-O complexity is derived for the individual algorithms (*BWO* and *GEO*) and for their hybrid variants (${\mathrm{GEO}-\mathrm{BWO}}^{HA}$, ${\mathrm{GEO}}^{PR-CR}$, and *GEO^PM^*) in order to formally characterize their computational growth behavior. The detailed complexity analysis for each case is presented in the following subsections.

#### 5.3.1. Computational Cost of the Fitness Evaluation

The objective function evaluation is one of the main computational components of the proposed implementation. In the implemented procedure, based on the parameters mentioned in Section 3.3.1, the selected route of each part is first transformed into a sequence, time, and cell structure. This requires scanning the machine information associated with each selected route, with a maximum cost of O(n·m).

After that, the operations of each selected route are sorted according to their production sequence to correctly identify intercellular movements. Since $K_{ij}\leq m$, the sorting process can be bounded in the worst case by O(n·mlogm), according to Eberl [61].

Finally, the intercellular movement cost is calculated by comparing consecutive operations in each selected route, which requires O(n·m) operations. The cost associated with reliability is also computed by scanning the processing information associated with each part and machine, requiring O(n·m) operations. Therefore, the total computational cost of the fitness evaluation for one solution is:

(22) $$F=O\left(n\cdot m+n\cdot m\log m+n\cdot m+n\cdot m\right).$$

By retaining the dominant term, the fitness evaluation cost is simplified as:

(23) F=O(n·mlogm).

#### 5.3.2. Computational Cost of the Repair Mechanism

The repair mechanism is applied when a generated or modified solution does not satisfy the feasibility conditions of the mathematical model. Based on the parameters mentioned in Section 3.3.1, the solution vector has a size of $N_{var}=n+m$. The first stage scans the complete solution vector to verify whether each element is within its feasible numerical domain. If an infeasible value is detected, it is replaced by a randomly generated feasible value within the corresponding lower and upper limits. Since each correction has a constant cost, this stage has a computational cost of $O(N_{var})$.

The second stage verifies the distribution of machines across cells. This requires extracting the last *m* elements of the vector and counting the number of machines assigned to each cell, with a cost of O(m+c). If the lower or upper capacity limits are not satisfied, one machine assignment is randomly corrected by assigning it to a feasible cell. Since this correction is also a constant-time operation, the cost of one verification and correction attempt is O(m+c).

Let ρ represent the number of repetitions required by the repair loop until all cell capacity limits are satisfied. Therefore, the repair cost can be represented as:

(24) $$R=O\left(N_{var}+\rho \cdot \left(m+c\right)\right).$$

#### 5.3.3. Computational Complexity of the BWO

Once the auxiliary costs of the fitness evaluation and repair mechanism have been defined, the computational complexity of the *BWO* can be derived from its initialization and iterative search stages. Let *T* denote the maximum number of iterations. The auxiliary costs of the fitness evaluation and the repair mechanism were previously defined in Equations (23) and (24), respectively.

The initialization stage generates the initial population, verifies feasibility, repairs solutions when necessary, evaluates fitness, and sorts the population. Therefore, its computational cost is:

(25) $$O\left(N_{pop}\cdot \left(N_{var}+R+F\right)+N_{pop}\log N_{pop}\right).$$

During each iteration, the procreation stage modifies approximately $N_{var}/2$ positions of the solution vector for each reproduction. Since constant factors are omitted in Big-O notation, this process has a cost of $O(nr\cdot N_{var})$. The children generated through procreation and the individuals modified by mutation are then verified, repaired if necessary, and evaluated. This adds a cost of O((nr+nm)·(R+F)). Finally, the population is updated by merging the current population, the surviving children, and the mutated individuals.

If *S* denotes the temporary population size after merging, with $S=O(N_{pop}+nr+nm)$, the sorting process requires O(SlogS). Since nr and nm are proportional to $N_{pop}$, and $S=O(N_{pop})$, the dominant computational complexity of the *BWO* can be expressed as:

(26) $$O\left(T\cdot N_{pop}\cdot \left(N_{var}+R+F\right)+T\cdot N_{pop}\log N_{pop}\right).$$

Substituting *R* and *F*, the final dominant complexity is:

(27) $$O\left(T\cdot N_{pop}\cdot \left[N_{var}+m+c+n\cdot m\log m\right]+T\cdot N_{pop}\log N_{pop}\right).$$

#### 5.3.4. Computational Complexity of the GEO

According to the computational analysis presented in the original description of the *GEO* [7], the initialization of the position vectors has a cost of $O(N_{pop}\cdot N_{var})$. However, in the proposed implementation, this stage also includes feasibility verification, possible repair, fitness evaluation, and sorting of the initial population. Therefore, the initialization cost is:

(28) $$O\left(N_{pop}\cdot \left(N_{var}+R+F\right)+N_{pop}\log N_{pop}\right).$$

During the main iterative loop, each solution updates its position through the attack and cruise mechanisms of the *GEO*. This process involves vector operations across all decision variables for each individual; therefore, the position update stage has a computational cost of $O(N_{pop}\cdot N_{var})$ per iteration.

After this update, the generated solutions are verified for feasibility, repaired when necessary, and evaluated through the objective function. Using the auxiliary costs previously defined in Equations (23) and (24), this adds the cost of fitness calculation and repair per iteration. Since these operations are performed during *T* iterations, the dominant computational complexity of the *GEO* can be expressed as:

(29) $$O\left(N_{pop}\cdot \left(N_{var}+R+F\right)+N_{pop}\log N_{pop}+T\cdot N_{pop}\cdot \left(N_{var}+R+F\right)\right).$$

By grouping common terms and substituting *R* and *F*, the final dominant complexity becomes:

(30) $$O\left(T\cdot N_{pop}\cdot \left[N_{var}+\rho \cdot \left(m+c\right)+n\cdot m\log m\right]+N_{pop}\log N_{pop}\right).$$

#### 5.3.5. Computational Complexity of the $GEO-BWO^{HA}$

In the $GEO-BWO^{HA}$, both metaheuristics operate sequentially within the same iterative process. Therefore, its computational complexity is obtained by combining the dominant terms of the complete *GEO* stage and the complete *BWO* stage, previously defined in Equations (30) and (27), respectively.

Since both stages work over the same population and use the same solution representation, the initialization is considered once, while the iterative cost includes the position update of *GEO* and the procreation, cannibalism, and mutation operators of *BWO*. Thus, the dominant complexity of the complete hybrid approach can be expressed as:

(31) $$O\left(T\cdot N_{pop}\cdot \left[N_{var}+\rho \cdot \left(m+c\right)+n\cdot m\log m\right]+T\cdot N_{pop}\log N_{pop}\right).$$

#### 5.3.6. Computational Complexity of the $GEO^{PR-CR}$

In the hybrid $GEO^{PR-CR}$ algorithm, the complete *GEO* procedure is combined only with the procreation and cannibalism stages of the *BWO*. Therefore, the additional cost with respect to *GEO* is associated with generating children, applying feasibility verification, repairing solutions when necessary, and evaluating the generated individuals.

Since the procreation and cannibalism stages operate over a fraction of the population and nr is proportional to $N_{pop}$, their contribution does not change the dominant order of the complete *GEO* procedure. Therefore, the overall complexity of $GEO^{PR-CR}$ is:

(32) $$O\left(T\cdot N_{pop}\cdot \left[N_{var}+\rho \cdot \left(m+c\right)+n\cdot m\log m\right]+T\cdot N_{pop}\log N_{pop}\right).$$

#### 5.3.7. Computational Complexity of the $GEO^{PM}$

In the *GEO^PM^* algorithm, the complete *GEO* procedure is combined with the mutation stage of the *BWO*. The mutation operator performs a positional swap between two elements of the solution vector. Although this swap has a constant cost, each mutated solution must be checked for feasibility, repaired when necessary, and evaluated through the objective function.

Since the number of mutated individuals nm is proportional to $N_{pop}$, the mutation stage adds a linear contribution with respect to the population size. As a result, the dominant order remains governed by the same repair and fitness evaluation terms. Thus, the overall complexity of *GEO^PM^* is:

(33) $$O\left(T\cdot N_{pop}\cdot \left[N_{var}+\rho \cdot \left(m+c\right)+n\cdot m\log m\right]+T\cdot N_{pop}\log N_{pop}\right).$$

## 6. Conclusions

This study addressed the GCFP-MR through the application and comparative assessment of metaheuristic approaches. Although the *BWO* was initially considered as a reference methodology, the results confirmed that alternative metaheuristics can provide competitive or superior performance when appropriately adapted to the problem structure.

The comparative analysis demonstrated that the *BWO* achieved better individual performance than the *GEO*, as reflected by lower RPD values. Specifically, *BWO* reached an average RPD of 0.855%, while *GEO* obtained 1.068%, indicating a more effective balance between exploration and exploitation in the search process.

For this investigation, three hybrid strategies were proposed by incorporating selected mechanisms from *BWO*. The first hybridization sequentially combined *GEO* and *BWO* within each iteration cycle ($GEO-BWO^{HA}$). The second hybridization integrated the procreation and cannibalism phases of *BWO* into the *GEO* framework to intensify solution refinement ($GEO^{PR-CR}$). The third hybridization focused on integrating the mutation phase of *BWO* after the execution of *GEO*, aiming to enhance diversification and reduce the risk of premature convergence (*GEO^PM^*).

The experimental results revealed that the effectiveness of hybridization strongly depends on the specific mechanism incorporated. Among the evaluated approaches, *GEO^PM^* achieved the best overall performance, reaching an RPD of 0.592%, followed by $GEO-BWO^{HA}$ (0.734%) and $GEO^{PR-CR}$ (1.162%). All the results were obtained from more than 4,110,720 experimental runs, providing a robust empirical basis for performance comparison.

To assess the statistical significance of the observed differences, WMW tests were conducted among the evaluated algorithms, including *GEO*, *BWO*, and the hybrid variants. The statistical analysis showed that *GEO^PM^* achieved statistically significant differences with respect to *GEO* and $GEO^{PR-CR}$. However, no statistically significant differences were observed between *GEO^PM^*, *BWO*, and $GEO-BWO^{HA}$. Therefore, the statistical evidence does not support the existence of a single statistically dominant algorithm, but rather a competitive group of high-performing methods.

Similarly, comparative graphical analyses were performed using box plots and convergence graphs. Box plots were used to summarize the distribution of the results obtained by each algorithm, while convergence graphs were included to illustrate how the best objective value evolves over iterations, allowing the convergence behavior of the algorithms to be observed. To ensure a fair and consistent evaluation, 300 random initial populations were generated, and each was solved using the best parameter configuration identified for each algorithm.

In addition, a computational complexity analysis based on Big-O notation was conducted for each metaheuristic and hybrid configuration. This analysis was included to provide a formal and comparative characterization of the theoretical computational structure of the proposed approaches, without aiming to establish empirical performance relationships or scalability conclusions.

Overall, the results indicate that algorithms exploiting the *BWO* mutation operator tend to produce better results, suggesting that successful hybridization does not necessarily depend on the fusion of complete metaheuristic structures. Rather, performance improvements can be achieved more effectively by selectively incorporating specific operators that enhance the search dynamics of the original method. Therefore, the proposed *GEO^PM^* can be considered a robust and competitive metaheuristic for the GCFP-MR, particularly due to its descriptive performance and its high frequency of reaching the optimal solution.

Future research directions include the explicit incorporation of machine repair dynamics by considering both MTBF and Mean Time To Repair (MTTR), enabling the direct modeling of machine availability. Furthermore, the integration of machine learning techniques for parameter adaptation and hybrid control, as well as the evaluation of the proposed approaches on larger and more heterogeneous industrial datasets, represent promising avenues for further research. It is important to note that the selected parameter configuration could be interpreted as the best-performing configuration for the analyzed case study, rather than as a universally optimal configuration. Therefore, future studies should consider a broader set of benchmark instances and adopt a separate tuning and validation procedure, in which parameter configurations are calibrated on one set of instances and evaluated on independent cases. This would reduce the risk of overfitting and provide stronger evidence regarding the robustness and generalizability of the proposed approaches.

## Figures and Tables

**Table 1:** Summary of selected GCFP-MR studies involving metaheuristics and hybridization strategies.

Year	Reference	Problem Variant	Metaheuristic Used	Hybridization	Case Study
2008	Jabalameli et al. [32]	GCFP-MR	SA, GA, MA with local search mechanism	Partially	Numerical examples compared with a branch-and-bound algorithm
2014	Jouzdani et al. [33]	GCFP-MR	Modified SA	No	Numerical examples used to evaluate the proposed method
2017	Karoum and Elbenani [34]	GCFP-MR	Modified CSA with local search mechanism	Partially	Numerical case compared with the branch-and-bound method
2019	Karoum and Elbenani [24]	GCFP-MR	CS with local search mechanism	Partially	Benchmark problems with various scales compared with SA
2019	Golmohammadi et al. [39]	GCFP	GA, KA, RDA	Yes	Numerical instances validated with an exact solver and a real case study
2023	Figueroa-Torrez et al. [25]	GCFP-MR	B-BWO	No	Benchmark case with 8 parts, 9 machines, and 20 routes compared with CS
2026	This study	GCFP-MR	*BWO*, *GEO* and hybrid variants	Yes	Comparative computational experiments with statistical validation

**Table 2:** Case study.

Sequence/ Processing Time (min)	Parts (Volume)																			
	P1 (75)			P2 (130)			P3 (110)		P4 (145)		P5 (110)		P6 (105)		P7 (140)				P8 (115)	
Machines	R1	R2	R3	R1	R2	R3	R1	R2	R1	R2	R1	R2	R1	R2	R1	R2	R3	R4	R1	R2
**M1**	1 (5)			1 (4)			1 (4)	1 (4)	1 (5)	1 (5)		1 (4)		1 (4)	1 (5)			1 (5)	1 (4)	
**M2**		1 (5)	1 (5)		1 (5)	1 (5)					1 (3)		1 (5)			1 (3)	1 (3)	2 (3)		1 (4)
**M3**							2 (3)	2 (3)	2 (3)	2 (3)										
**M4**	2 (3)			2 (4)				3 (3)	3 (5)	3 (5)		2 (4)			2 (4)		2 (4)			
**M5**	3 (4)		2 (4)	3 (3)	2 (3)	2 (3)	3 (4)			4 (4)					3 (3)		3 (3)			
**M6**		2 (5)														2 (4)		3 (4)	2 (3)	2(4)
**M7**											2 (5)	3 (5)	2 (5)	2 (5)						
**M8**		3 (4)		4 (4)		3 (4)	4 (3)	4 (3)	4 (3)	5 (4)			3 (5)	3 (5)			4(5)			
**M9**	4 (5)		3 (5)		3 (3)						3 (5)	4 (5)			4 (5)	3 (5)		4 (5)		
**Intercellular cost**	**375**	**375**	**0**	**1300**	**0**	**650**	**1100**	**0**	**0**	**1450**	**1100**	**550**	**525**	**0**	**700**	**0**	**700**	**700**	**575**	**0**

**Table 3:** MTBF and BC.

Machine	MTBF ^1^ (h)	BC ^2^
M1	90	900
M2	51	2000
M3	73	2000
M4	60	1600
M5	76	1500
M6	62	1800
M7	71	1400
M8	58	1700
M9	65	1500

^1^ Mean time between failure; ^2^ Breakdown cost.

**Table 4:** Best RPD values obtained with *BWO*.

$N_{\mathrm{pop}}$	*Iter*	*PR*	*CR*	*PM*	*RPD*
100	50	0.8	0.8	0.8	0.855%
100	50	0.8	0.8	0.6	0.880%
100	75	0.8	0.4	0.6	0.995%
75	75	0.8	0.8	0.6	1.016%
100	50	0.8	0.6	0.8	1.038%
100	75	0.6	0.4	0.4	1.046%
100	75	0.8	0.4	0.4	1.054%
100	100	0.8	0.6	0.8	1.058%
100	75	0.8	0.6	0.6	1.086%
50	75	0.8	0.4	0.6	1.088%

*Note:* Darker green background colors indicate lower RPD values and, therefore, better-performing configurations.

**Table 5:** Best RPD values obtained with *GEO*.

$N_{\mathrm{pop}}$	*Iter*	$p_{a}$	$p_{c}$	*RPD*
100	100	[1.5, 2.0]	[1.00, 0.25]	1.068%
100	75	[1.5, 2.0]	[1.00, 0.25]	1.099%
100	100	[0.0, 2.0]	[1.00, 0.25]	1.142%
100	50	[1.5, 2.0]	[1.00, 0.00]	1.147%
100	100	[1.5, 2.0]	[1.00, 0.00]	1.157%
100	100	[1.5, 2.0]	[1.00, 0.75]	1.159%
100	100	[1.5, 2.0]	[1.00, 0.50]	1.162%
100	75	[1.0, 2.0]	[1.00, 0.75]	1.164%
100	100	[1.0, 2.0]	[1.00, 0.00]	1.183%
100	50	[1.5, 2.0]	[0.75, 0.50]	1.186%

*Note:* Darker green background colors indicate lower RPD values and, therefore, better-performing configurations.

**Table 6:** Best RPD values obtained with $GEO-BWO^{HA}$.

$N_{\mathrm{pop}}$	*Iter*	$p_{a}$	$p_{c}$	*PR*	*CR*	*PM*	*RPD*
100	50	[0.5, 1.5]	[1.00, 0.50]	0.8	0.4	0.8	0.734%
100	75	[0.0, 2.0]	[1.00, 0.50]	0.8	0.6	0.8	0.761%
100	75	[0.0, 0.5]	[0.75, 0.25]	0.8	0.4	0.4	0.766%
100	50	[0.5, 1.0]	[1.00, 0.00]	0.6	0.8	0.8	0.771%
100	75	[0.0, 0.5]	[1.00, 0.25]	0.8	0.8	0.8	0.773%
100	50	[1.5, 2.0]	[1.00, 0.50]	0.8	0.8	0.8	0.778%
100	75	[1.5, 2.0]	[1.00, 0.25]	0.4	0.4	0.8	0.787%
100	75	[1.0, 2.0]	[0.75, 0.00]	0.8	0.6	0.8	0.792%
100	75	[0.5, 2.0]	[1.00, 0.00]	0.4	0.8	0.6	0.798%
100	100	[0.5, 1.0]	[0.75, 0.00]	0.8	0.4	0.6	0.800%

*Note:* Darker green background colors indicate lower RPD values and, therefore, better-performing configurations.

**Table 7:** Best RPD values obtained with ${\mathrm{GEO}}^{PR-CR}$.

$N_{\mathrm{pop}}$	*Iter*	$p_{a}$	$p_{c}$	*PR*	*CR*	*RPD*
100	50	[1.5, 2.0]	[0.50, 0.25]	0.8	0.6	1.162%
100	75	[1.5, 2.0]	[1.00, 0.50]	0.8	0.8	1.191%
100	75	[1.5, 2.0]	[0.75, 0.50]	0.8	0.4	1.191%
100	50	[0.0, 2.0]	[0.75, 0.50]	0.6	0.6	1.210%
100	50	[0.0, 1.5]	[0.75, 0.50]	0.8	0.8	1.210%
100	75	[1.5, 2.0]	[0.75, 0.25]	0.6	0.8	1.231%
100	50	[1.5, 2.0]	[1.00, 0.75]	0.6	0.4	1.232%
100	50	[1.0, 2.0]	[1.00, 0.75]	0.6	0.6	1.239%
100	75	[1.5, 2.0]	[1.00, 0.75]	0.8	0.8	1.244%
100	100	[0.0, 0.5]	[0.25, 0.00]	0.8	0.4	1.248%

*Note:* Darker green background colors indicate lower RPD values and, therefore, better-performing configurations.

**Table 8:** Best RPD values obtained with ${\mathrm{GEO}}^{PM}$.

$N_{\mathrm{pop}}$	*Iter*	$p_{a}$	$p_{c}$	*PM*	*RPD*
100	100	[0.5, 2.0]	[0.50, 0.00]	0.8	0.592%
100	75	[0.5, 1.0]	[0.75, 0.50]	0.6	0.648%
100	100	[0.5, 1.0]	[0.50, 0.25]	0.6	0.649%
100	100	[0.0, 2.0]	[1.00, 0.25]	0.6	0.672%
100	100	[0.5, 1.0]	[1.00, 0.00]	0.6	0.674%
75	50	[0.5, 1.0]	[0.75, 0.00]	0.8	0.683%
100	100	[0.0, 1.0]	[1.00, 0.50]	0.4	0.684%
100	75	[0.5, 2.0]	[0.75, 0.00]	0.6	0.685%
100	100	[0.5, 1.0]	[0.75, 0.50]	0.4	0.693%
100	100	[0.0, 1.0]	[1.00, 0.50]	0.6	0.698%

*Note:* Darker green background colors indicate lower RPD values and, therefore, better-performing configurations.

**Table 9:** Wilcoxon–Mann–Whitney results among the five algorithms.

	BWO	GEO	$\mathrm{GEO}-{\mathrm{BWO}}^{\mathrm{HA}}$	${\mathrm{GEO}}^{\mathrm{PR}-\mathrm{CR}}$	${\mathrm{GEO}}^{\mathrm{PM}}$
** BWO **		**<0.05**	≥0.05	**<0.05**	≥0.05
** GEO **	≥0.05		≥0.05	**<0.05**	≥0.05
** $GEO-BWO^{HA}$ **	≥0.05	**<0.05**		**<0.05**	≥0.05
** $GEO^{PR-CR}$ **	≥0.05	≥0.05	≥0.05		≥0.05
** $GEO^{PM}$ **	≥0.05	**<0.05**	≥0.05	**<0.05**	

Green: p<0.05; Red: p≥0.05; Gray: not applicable.

## Data Availability

Data available on request from the authors. The data that support the findings of this study are available from the corresponding author, O.D., upon reasonable request.
